# Chemical tunnel-splitting-engineering in a dysprosium-based molecular nanomagnet

**DOI:** 10.1038/s41467-018-03706-x

**Published:** 2018-03-29

**Authors:** Mikkel A. Sørensen, Ursula B. Hansen, Mauro Perfetti, Kasper S. Pedersen, Elena Bartolomé, Giovanna G. Simeoni, Hannu Mutka, Stéphane Rols, Minki Jeong, Ivica Zivkovic, Maria Retuerto, Ana Arauzo, Juan Bartolomé, Stergios Piligkos, Høgni Weihe, Linda H. Doerrer, Joris van Slageren, Henrik M. Rønnow, Kim Lefmann, Jesper Bendix

**Affiliations:** 1 Department of Chemistry, University of Copenhagen, 2100 Copenhagen, Denmark; 2 Institut für Physikalische Chemie, Universität Stuttgart, Pfaffenwaldring 55, 70569 Stuttgart, Germany; 3 Niels Bohr Institute, University of Copenhagen, 2100 Copenhagen, Denmark; 4 Laboratory for Quantum Magnetism, École Polytechnique Fédérale Lausanne, 1015 Lausanne, Switzerland; 5 Escola Universitària Salesiana de Sarrià (EUSS), Passeig Sant Joan Bosco 74, 08017 Barcelona, Spain; 6 Forschungsneutronenquelle Heinz Maier-Leibnitz FRM II, Technische Universität München, 85748 Garching, Germany; 7 Institute Laue–Langevin, BP 156, 38042 Grenoble Cedex 9, France; 8 University of Zaragoza, CSIC-Instituto de Cìencia de Materiales de Aragón (ICMA), Pedro Cerbuna 12, 50009 Zaragoza, Spain; 9 Department of Chemistry, Boston University, Boston, MA 02215 USA; 10 Present Address: Department of Chemistry, University of Copenhagen, 2100 Copenhagen, Denmark; 11 Present Address: Department of Chemistry, Technical University of Denmark, 2800 Lyngby, Denmark; 12 Present Address: Institute of Aerospace Thermodynamics, Universität Stuttgart, Pfaffenwaldring 31, 70569 Stuttgart, Germany; 13 Present Address: Instituto de Catálisis y Petroleoquímica – CSIC, 28049 Madrid, Spain

## Abstract

Total control over the electronic spin relaxation in molecular nanomagnets is the ultimate goal in the design of new molecules with evermore realizable applications in spin-based devices. For single-ion lanthanide systems, with strong spin–orbit coupling, the potential applications are linked to the energetic structure of the crystal field levels and quantum tunneling within the ground state. Structural engineering of the timescale of these tunneling events via appropriate design of crystal fields represents a fundamental challenge for the synthetic chemist, since tunnel splittings are expected to be suppressed by crystal field environments with sufficiently high-order symmetry. Here, we report the long missing study of the effect of a non-linear (*C*_4_) to pseudo-linear (*D*_4d_) change in crystal field symmetry in an otherwise chemically unaltered dysprosium complex. From a purely experimental study of crystal field levels and electronic spin dynamics at milliKelvin temperatures, we demonstrate the ensuing threefold reduction of the tunnel splitting.

## Abstract (web summary)

Suppression of quantum tunneling in molecular magnets is key for their magnetic behaviours to be exploitable. Here, the authors show that tuning the geometry of lanthanide single-ion magnets leads to a suppression of the quantum tunneling, finding a three-fold reduction of the tunnel splitting upon changing the crystal field symmetry.

## Introduction

Quantum tunneling of the magnetization is one of the key quantum signatures of the class of molecular nanomagnets commonly known as single-molecule magnets^1^. The phenomenon is observed in the bulk of polynuclear 3d metal clusters^2,3^ and mononuclear^4–6^ or multinuclear^7–9^ lanthanide complexes, but can also be observed for single molecules deposited on inorganic surfaces^10^ or incorporated into nanodevices^11^. Indeed, the quantum nature of these molecular objects renders them promising building units for molecular spintronics^12,13^, quantum computing^14,15^, and high-density information storage^12,16^. Advances have been made in these areas as exemplified by the realization of a nuclear spin qubit transistor^11^, and a supramolecular spin valve^17^; with both devices relying on the terbium-based molecular nanomagnet [Tb(pc)_2_] (pc = phtalocyaninate). Recently, it was demonstrated that tunneling of the electronic spins in [Tb(pc)_2_] can be suppressed at the molecular level, by coupling the molecule to a carbon nanotube resonator with an appropriately discrete phonon spectrum^18^.

Suppression of tunneling in the bulk has been a key target in the design of new molecular nanomagnets, especially those based on single lanthanide ions^19^. This desire arises from the wish to enhance the superparamagnet-like behavior of this class of molecules^20^ resulting from thermally activated spin-lattice relaxation (SLR) known as Orbach relaxation^21^ (a resonant spin–phonon interaction involving an excited crystal field (CF) state). Indeed, a free lanthanide ion exhibits strong spin–orbit coupling and has therefore a Russell–Saunders (RS) ground multiplet characterized by a total angular momentum *J*, and the effect of a CF is to lift the degeneracy of the |*J*,*m*_*J*_〉 substates of the multiplet, the total energy splitting usually being on the order of 10^2^–10^3^ cm^−1^.

The energy separation between the ground and excited states can be regarded as a barrier for spin reversal, equivalent to the anisotropy barrier of superparamagnetic nanoparticles^22^. However, at low temperatures, quantum tunneling eclipses the Orbach relaxation^20^. As a result, tunneling becomes an obstacle to applications relying on the magnet-like behavior of these molecular nanomagnets (e.g., high-density information storage). The ground state tunnel splitting is created by low-symmetry components of the CF, described by off-diagonal terms in the associated CF Hamiltonian. Consequently, many chemists started to synthetize compounds encapsulating lanthanides in different geometries and study their influence on the magnetic properties^23–25^. Major results have been achieved targeting CF environments with high-order symmetry axes commanding an effective linear CF Hamiltonian (diagonal in a symmetry-defined basis^19^), thus efficiently suppressing the tunnel splitting. Results obtained for *D*_4d_^26–28^ and *D*_5h_^29–31^ symmetries indicate this to be a viable strategy, but a true comparative study demonstrating the minimization of off-diagonal terms in the CF Hamiltonian when changing from non-linear to a pseudo-linear point group symmetry is still missing. So far, the few reported studies either suffer from the lack of chemical comparability between the studied species^19^, or the change in coordination number of the lanthanide ion^32^. Indeed, the realization of lanthanide coordination complexes for which the point symmetry can be tuned, while leaving the complex chemically unaltered is far from trivial. Some of us recently reported on a family of complexes possessing exactly this property^33^. Here, we report on the CF quantification and the electronic spin dynamics of the dysprosium derivatives of this family, demonstrating and quantifying the effect of an increase in point group symmetry (from non-linear *C*_4_ to pseudo-linear idealized *D*_4d_ within a 1 % error bar) on the ground state tunnel splitting for an otherwise unaltered lanthanide complex.

## Results

### Molecular structures

**Fig. 1:**
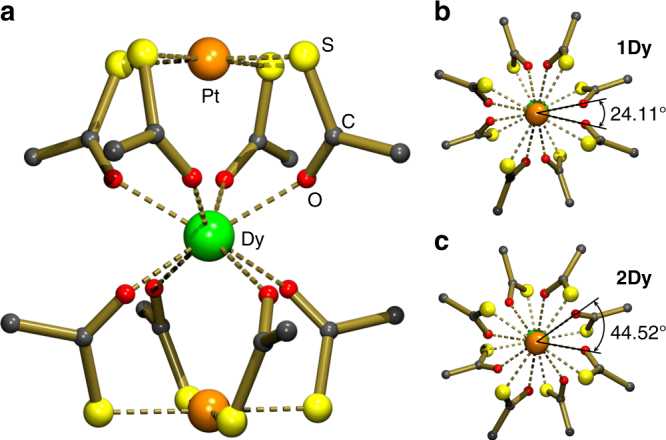
Molecular structures. Molecular structure of the anionic dysprosium complex in crystals of **2Dy**^33^ viewed perpendicular to the *c* axis (**a**). **1Dy**^33^ (**b**) and **2Dy** (**c**) viewed along the fourfold axis (*c* axis of the unit cell), with the respective values of the twist angle given

We recently demonstrated the possibility of exerting a high degree of control over lanthanide CF environments by relying on a combination of HSAB (hard–soft-acid–base) preferences and the predictable square-planar geometries of group 10 metal complexes acting as ligands^33^. Usage of [M^II^(SAc)_4_]^2−^ (M = Pd, Pt; SAc^−^ = thioacetate) metalloligands towards lanthanide ions results in fourfold symmetrical [Ln{M(SAc)_4_}_2_]^−^ complexes, which, when combined with symmetry-compatible counter cations, form crystals containing the lanthanide ion at a site of crystallographically imposed fourfold symmetry. For Ln = Dy, we reported the structures of the salts [NEt_4_][Dy{Pt(SAc)_4_}_2_] (**1Dy**, space group: *P*4/*mcc*)^33^, and [PPh_4_][Dy{Pt(SAc)_4_}_2_] (**2Dy**, space group: *P*4/*n*)^33^. The structure of the anionic dysprosium complex in crystals of the latter salt is given in Fig. 1a. Upon comparing the two compounds, the change of counter cation induces a simple yet crucial structural change. We identify two sets of four coordinating oxygen atoms, stemming from the metalloligands, each describing the vertices of a perfect square. The two squares (above and below the dysprosium ion) originate two parallel planes perpendicular to the *C*_4_ axis. The twist angle between the diagonals of the two squares constitutes the only difference between the two complexes and is 24.11° and 44.52° for **1Dy** and **2Dy**, respectively (Fig. 1b,c). Through a detailed electron paramagnetic resonance (EPR) study of the zero-field splittings of the weakly anisotropic, isometric Gd^3+^ derivatives, the respective *C*_4_ and approximate, but almost perfect *D*_4d_ point symmetries of the **1Ln** and **2Ln** derivatives inferred from structural metrics were verified to hold true from an electronic point of view^33^. We emphasize that **2Dy** adopts idealized *D*_4d_ symmetry to a very good approximation (a twist angle of 44.52° vs. the ideal 45°, for which a diagonal CF Hamiltonian is expected, and the eigenstates therefore would be pure |*J*,*m*_*J*_〉 states^34^). Due to their extremely similar chemical structures, **1Dy** and **2Dy** represent perfect test subjects for a study of how the tunnel splitting can be controlled via chemical engineering of the point group symmetry.

### Crystal field quantification

**Fig. 2:**
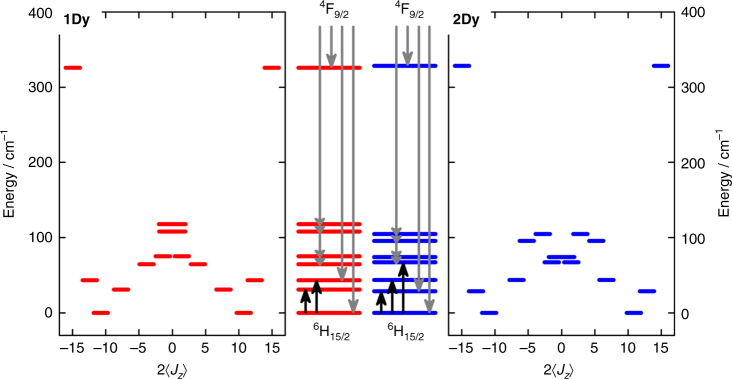
Crystal field splittings. Energy versus 2〈*J*_*z*_〉 for the crystal field states of the ground ^6^H_15/2_ multiplet for **1Dy** and **2Dy** corresponding to the CF parameters in Table 1. In the center panel, showing the eight Kramers doublets, the black arrows denote the observed INS transitions, while the grey arrows denote the individual transitions observed for the ^4^F_9/2_→^6^H_15/2_ emission line. Of the latter type, the multi-headed arrows denote the four excited doublets fully or partially assigned to the broad maximally intense band in the luminescence spectra of **1Dy** (Fig. 3a), and **2Dy** (Fig. 3b)

**Fig. 3:**
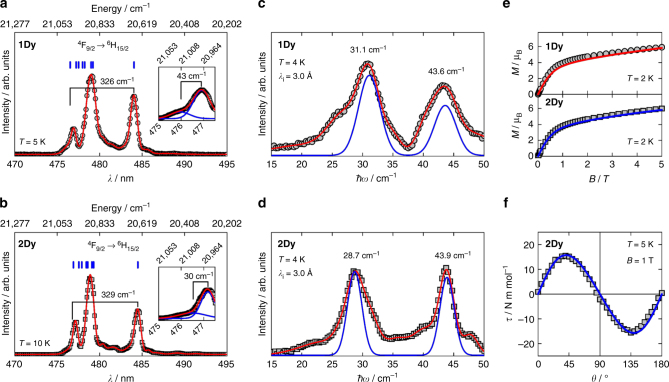
Crystal field quantification. Low-temperature luminescence spectra (^4^F_9/2_→^6^H_15/2_) for **1Dy** (**a**, *T* = 5 K) and **2Dy** (**b**, *T* = 10 K) recorded with excitation wavelengths of *λ*_ex_ = 386 and 387 nm, respectively. For **1Dy** (**2Dy**) the red line is a fit to a sum of 9 (11) Gaussians, and the vertical blue lines denote the positions of the Kramers doublets within the ^6^H_15/2_ ground multiplet. The insets give high-resolution spectra of the two peaks at highest energy, with best fits to a sum of two Gaussians. INS spectra (integrated over 0.95 Å^−1^ ≤ *Q* ≤ 1.8 Å^−1^) for **1Dy**(**c**) and **2Dy** (**d**) at* T* = 4 K recorded with an incident neutron wavelength of *λ*_i_ = 3.0 Å. For **1Dy** (**2Dy**) the red line is a fit to a sum of 7 (8) Gaussians. The blue lines are the simulated INS spectra based on CF parameters in Table 1. In **c** and **d**, the errors are less than the size of the symbols. Powder-averaged molar magnetization for **1Dy** (**e**, top) and **2Dy**(**e**, bottom) at *T* = 2 K, with lines indicating the best fits. Molar magnetic torque of a single crystal of **2Dy** (**f**) at *T* = 5 K recorded in an applied magnetic field of *B* = 1 T. *θ* denotes the angle (increasing anticlockwise) between the crystallographic ab plane and the applied magnetic field. The blue line represents the angular dependence of the magnetic torque simulated from the CF parameters for **2Dy** given in Table 1

The calculation of tunnel splittings in lanthanide complexes requires an intimate knowledge of the CF energy levels and the associated CF state compositions. In the present work, the CF levels of **1Dy** and **2Dy** (Fig. 2) have been experimentally determined from a combination of luminescence spectroscopy (Fig. 3a,b), inelastic neutron scattering (INS, Fig. 3c,d), and magnetization measurements (Fig. 3e and Supplementary Fig. 1). The large RS intermultiplet separation for dysprosium (Δ*E*(^6^H_15/2_−^6^H_13/2_) ~ 3000 cm^−1^)^34^, allows us to safely neglect the mixing of the ground multiplet with excited ones. Hence, we use the Stevens equivalent operator approach, with the CF potential expanded as a sum of equivalent angular momentum operators, operating exclusively on the ground *J* = 15/2 multiplet^35^. According to the tetragonal symmetry of the systems, the Hamiltonian is (Methods section)

1 $$\hat H = \hat H_{{\mathrm{Zeeman}}} + \hat H_{{\mathrm{CF}}} = g_J\mu _{\mathrm{B}}{\mathbf{B}} \cdot {\hat{\mathbf J}} + \mathop {\sum}\limits_{k = 2,4,6} {B_k^0} \hat O_k^0 + B_4^4\hat O_4^4$$

in which the first term accounts for the electronic Zeeman interaction, and the latter terms for CF effects. The four CF parameters *B*_*k*_^*q*^ have been determined from the combination of the above-mentioned spectroscopic observations, and the previously reported powder magnetization and magnetic susceptibility data^33^.

Here we discuss the best-fit parameters, as reported in the Table 1. As for the diagonal components, we highlight their pronounced invariance (especially *B*_2_^0^ and *B*_4_^0^, varying less than 10 %) upon changing the twist angle. This is in accordance with our findings for the analogous gadolinium compounds^33^ and validates the choice of the model. The *B*_4_^4^*Ô*_4_^4^ term (off-diagonal in the |*J*,*m*_*J*_〉 = |15/2,*m*_*J*_〉 ≡ |*m*_*J*_〉 basis) accounts for the non-linear symmetry components of the CF, and is expected to vanish in the case of pseudo-linearity^19^. In our case, with near-perfect *D*_4d_ symmetry, the value of *B*_4_^4^ is reduced to about half upon going from **1Dy** to **2Dy**. The stronger off-diagonal contribution to the CF splitting in **1Dy** is evidenced in the strong admixing of excited |*m*_*J*_〉 states (experimentally derived compositions are given in Supplementary Tables 1 and 2). For **2Dy**, the eigenstate compositions are generally closer to pure |*m*_*J*_〉 states than for **1Dy**, yet again reflecting the higher symmetry of the former compound.

**Table 1:** Crystal field parameters^a^

	1Dy	2Dy
*g*_*J*_	4/3 (fixed)	4/3 (fixed)
*B*_2_^0^/cm^−1^	0.766(3)	0.802(4)
*B*_4_^0^/cm^−1^	7.126(6) × 10^−3^	6.60(1) × 10^−3^
*B*_6_^0^/cm^−1^	3.66(1) × 10^−5^	4.81(1) × 10^−5^
*B*_4_^4^/cm^−1^	11.27(9) × 10^−3^	7.0(2) × 10^−3^

^a^From a fit of luminescence, INS, and powder magnetization data to Eq. (1)

We shall now detail the spectroscopic observations serving as basis for the CF quantification. In the case of luminescence spectroscopy, we focused on the ^4^F_9/2_ → ^6^H_15/2_ emission line, known to allow the extraction of the CF splitting of the ground ^6^H_15/2_ RS multiplet of Dy(iii)^36,37^. In certain favorable cases, the position of all substates within the ground multiplet are observed^38^. That low-temperature emission line is notably similar for **1Dy** and **2Dy** (Fig. 3a,b), although the **1Dy** spectrum exhibits a significantly larger linewidth. This difference can be attributed to the presence of two slightly different CF environments in **1Dy** resulting from the disorder of the thioacetates, previously observed as giving rise to a linewidth difference in the X-band EPR spectra of the gadolinium analogues^33^. The excitation spectrum of **1Dy** at *T* = 5 K recorded with *λ*_em_ = 575 nm (Supplementary Fig. 2) facilitates the assignment of the high-energy shoulder positioned at 475.94(3) nm in the luminescence spectrum as the zero-phonon line. Similarly, we assign the corresponding band in the luminescence spectrum of **2Dy** (*T* = 10 K) positioned at 476.49(2) nm to be associated with the lowest level within the ground multiplet. Considering only the most intense sharp bands, the total splitting of the ^6^H_15/2_ multiplet is determined to be 326(3) cm^−1^ and 329(5) cm^−1^, for **1Dy** and **2Dy** respectively. Low-temperature high-resolution data on the two shortest wavelength bands yield splittings of 43(1) cm^−1^ and 30(14) cm^−1^ for **1Dy** and **2Dy**, respectively (insets of Fig. 3a,b).

INS experiments allowed for the high-resolution investigation of the CF excitations^39–41^. Since neutrons carry a magnetic moment (giving rise to the selection rule Δ*m*_*J*_ = ±1) and show direct interaction with both nuclear and magnetic degrees of freedom, assignment of CF excitations depend on analysis of momentum transfer (*Q*). The *Q*-integrated INS spectra recorded on polycrystalline samples of **1Dy** and **2Dy** at *T* = 4 K with an incident wavelength of *λ*_i_ = 3.0 Å are given in Fig. 3c,d. Two magnetic excitations positioned at energy transfers of 31.1 cm^−1^ and 43.6 cm^−1^ for **1Dy**, and 28.7 cm^−1^ and 43.9 cm^−1^ for **2Dy**, are clearly identified when compared with the pure phonon spectra of the isomorphous diamagnetic yttrium complexes, **1Y** and **2Y** (Supplementary Fig. 3). Their dispersionless nature (Supplementary Fig. 4) and the temperature dependence of the *Q*-integrated intensities (Supplementary Fig. 5) unambiguously identify them as CF excitations originating from the ground CF sublevel. We note that although the main contribution to the ground doublet is |±11/2〉, the |±3/2〉 contamination in **1Dy** is twice that in **2Dy**, due to the substantial difference in the magnitude of the *B*_4_^4^ CF parameter. Within an effective *J* = 1/2 approach this difference in ground state purity is reflected in an approximate threefold increase of the transverse component of ground state **g**-tensor upon decreasing the point group symmetry: **1Dy**, *g*_||_ = 14.38, *g*_┴_ = 0.226; **2Dy**, *g*_||_ = 14.53, *g*_┴_ = 0.081 (we recall that for a pure *m*_*J*_ = 11/2 state one has *g*_||_ = 14.67, *g*_┴_ = 0). The observed CF excitations are those corresponding to the first and second excited Kramers doublets, as their respective predominant |±9/2〉 and |±13/2〉 characters allow transitions from the ground state to be observed by INS (Δ*m*_*J*_ = ±1). Moreover, the INS spectra confirm the predictions of the simulated spectra based on the CF model (Fig. 3c,d, see Methods): due to the difference in geometry and effective symmetry, an inversion of the state identities between **1Dy** and **2Dy** must be considered when assigning the CF excitations. For **1Dy**, the first excited doublet is predominantly |±9/2〉, whilst for **2Dy**, the first excited doublet is essentially pure |±13/2〉. The integrated INS intensity of the higher-lying CF excitation, relative to the lower-lying one, unambiguously confirms this compositional difference. To complete the INS characterization (see Supplementary Figs 6 and 7), we note the presence of a weak CF excitation close to 67 cm^−1^ observed in **2Dy** with different experimental conditions, corresponding to the excitation from the ground doublet to the third excited doublet. This excitation is allowed due to the |±9/2〉 contribution to the wavefunction of the third excited state, with the moderate 25 % contribution yielding the observed low intensity.

The overall high purity of the ground states for **1Dy** and **2Dy**, implied by the derived CF models, finds further support in the absence of powder X-band EPR spectra at low temperatures (*T* < 15 K) for dilutions of the complexes in their respective yttrium hosts.

Single crystal cantilever torque magnetometry measurements on **2Dy** (Supplementary Figs 8–10) provide further validation of our CF model, especially the nature of the ground doublet^42^. Indeed, the torque simulated from the CF parameters given in Table 1 perfectly reproduces the experimental data at low temperatures (Fig. 3f), as well as at higher temperatures (Supplementary Fig. 8). Due to the presence of a single magnetic entity in the crystal structure, the sign of the torque unequivocally identifies the fourfold axis in **2Dy** as the easy axis of magnetization^43^, in agreement with the high *m*_*J*_ value of the ground state (i.e., *m*_*J*_ = 11/2). Additionally, the molar magnetization (Fig. 3e) and molar magnetic susceptibilities (Supplementary Fig. 1) of powder samples are also reproduced by the model.

It should be emphasized that the experimentally determined CF models (associated with the parameters listed in Table 1) are able to reproduce all spectroscopic (luminescence, INS) and thermodynamic (torque, magnetization, magnetic susceptibility) data. Yet again, we stress that the intimate knowledge of the CF splitting is a prerequisite for any meaningful assessment of the symmetry effects on the alteration of the time dependent properties of the dysprosium electronic spins. Furthermore, with such a detailed, experimentally based CF model at hand, **1Dy** and **2Dy** stand out as perfect systems for benchmarking state of the art computational approaches.

### Electronic spin relaxation

**Fig. 4:**
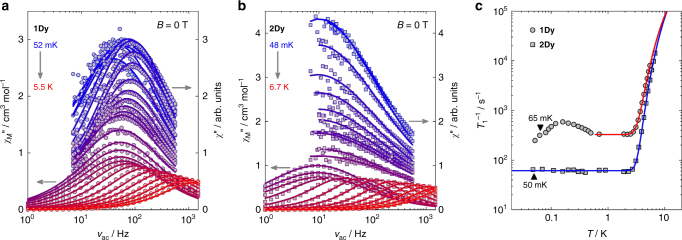
Electronic spin relaxation. Frequency dependence of the out-of-phase component of the ac magnetic susceptibility at zero applied dc field for **1Dy** (**a**), and **2Dy** (**b**) in the mK temperature range (right-hand ordinate) and at standard liquid helium temperatures (left-hand ordinate). The solid lines are best fits to Cole–Cole functions. The electronic spin-lattice relaxation (SLR) rates *T*_1_^−1^ for **1Dy** and **2Dy** (**c**) extracted from Cole–Cole fitting of the data in  **a** and  **b**. The solid lines are best fits to Eq. (2), and the denoted temperatures correspond to the estimated Néel temperatures

The electronic spin relaxation has been investigated via ac magnetic susceptibility, as typical relaxation times are of the order of milliseconds. In the temperature range 0.048–6.7 K, the response is strongly temperature and frequency dependent, and the non-zero imaginary component evidences spin dynamics on the timescale of the ac susceptibility experiment (Fig. 4a,b for **1Dy** and **2Dy**, respectively). In the present case, the two components of the susceptibility (i.e., the real and the imaginary one) are well-described by the Cole–Cole relations^44^ (Supplementary Figs 11–15 and Supplementary Note 1), giving direct access to the SLR rates. The temperature dependence of the SLR rate  is generally described as a sum of several processes. To avoid overparametrization, the minimum amount of relaxation mechanisms that satisfactorily reproduced the experimental data with physical meaningful parameters was used (vide infra). As commonly observed for Dy(iii) complexes^45,46^, our experimental data (Fig. 4c) can be well described by contributions from tunneling and Orbach relaxation processes^47^

2 $$T_1^{ - 1}(T) = \Gamma + \frac{{3M_{\mathrm{O}}^2{\Delta }^3}}{{\pi \hbar ^4\rho \nu ^5(\exp ({\Delta}/k_{\mathrm{B}}T) - 1)}}$$

where Γ is the tunneling rate, and the second term accounts for Orbach relaxation. Here, *M*_O_^2^ depends on the matrix elements of the relevant dynamic CF Hamiltonian over the states involved in the relaxation process (Supplementary Note 2), *Δ* is the separation between said states, *ρ* the crystal density determined from single crystal X-ray diffraction^33^, and *ν* the speed of the resonant phonon. If the latter quantity is approximated by the mean phonon velocity (*ν* = *v*_m_) in the relevant lattice (as determined from heat capacity measurements, Supplementary Fig. 16), the best-fit parameter values for **1Dy** (**2Dy**) are Γ = 327(2) s^−1^ (Γ = 63(4) s^−1^), *Δ* = 19.9(2) cm^−1^ (*Δ* = 25.8(4) cm^−1^), and *M*_O_^2^ = 2.8(1) × 10^2^ cm^−2^ (*M*_O_^2^ = 2.1(2) × 10^2^ cm^−2^).

As for the fitted values, we point out that the determined values of *M*_O_^2^ are of the expected magnitude^47^, but different for the two compounds. The dynamic CF potential can be expressed in terms of equivalent operators, in a fashion analogous to that adopted to express the static CF Hamiltonian. The nature of the microscopic mechanism responsible for the phonon-induced CF excitations leads to distinctly different dynamic effective CF Hamiltonians (Supplementary Note 2), and represents an independent support for the excited state inversion in the CF splittings of **1Dy** and **2Dy**. The determined value of *Δ* of 25.8(4) cm^−1^ for **2Dy** is in good agreement with the spectroscopic value of 28.7 cm^−1^ obtained from INS, the slightly reduced value obtained from the Orbach fit being the common result as the phonon-induced CF fluctuations produce a distribution of *Δ* values throughout the crystal^48^. Conversely, for **1Dy**, the determined value of *Δ* amounts to only 64% of the spectroscopic value. However, the field dependence of the SLR rate reveals that a tunneling contribution is present even at temperatures as high as *T* = 5 K (Supplementary Figs 17 and 18), which, however, can be quenched in an external field of *B* = 120 mT where the temperature dependence of *T*_1_^−1^ conforms perfectly to pure Orbach relaxation with *Δ* = 26.1(2) cm^−1^ (Supplementary Figs 19 and 20), in good agreement with the value obtained from INS. Based on this analysis, we can conclude that the SLR in zero external field is dominated by a resonant spin–phonon process involving the first excited doublet (i.e., an Orbach process) above ~4 K, while ground state tunneling dominates the dynamics below this temperature. The fivefold decrease in the tunneling rate for **2Dy** as compared to **1Dy** goes along with the close approach to *D*_4d_ symmetry and concomitant electronic linearity in the former system (vide infra).

As for the origin of the relaxation, the ac susceptibility measurements on **1Dy** diluted at a nominal 5% molar concentration into **1Y** reveals a complete quenching of the tunneling component in *T*_1_^−1^(*T*) at standard liquid helium temperatures (Supplementary Figs 21 and 22). The relaxation is of pure Orbach character, and the associated parameters are in good agreement with those of the concentrated species. This observation points to dipolar interactions with neighboring molecules as the dominant source of tunnel-splitting-opening transverse fields in the samples (vide infra). Consequently, a modified version of Prokof’ev–Stamp theory^5,6,49,50^ should apply, in which each individual dysprosium spin is viewed as embedded in a bath of environmental spins (i.e., the electronic spins residing on neighboring dysprosium centers). A Curie–Weiss analysis of the isothermal susceptibilities derived from Cole–Cole modeling suggests the average interactions between dysprosium centers to be antiferromagnetic in nature (**1Dy**: *θ* = –0.40(3) K, **2Dy**: *θ* = –0.30(8) K, Supplementary Fig. 23). Consequently, the critical temperatures for long-range magnetic ordering are determined from the inflection points of the lowest frequency *χ´*(*T*) curves^51,52^ (Supplementary Figs 24 and 25) resulting in Néel temperatures (*T*_N_) of 65 mK and ~50 mK for **1Dy** and **2Dy**, respectively. In the low-temperature regime above *T*_N_, these weak spin-spin interactions will give rise to a distribution of dynamic dipolar fields^49^. The longitudinal (*B*_dip,||_) and transverse (*B*_dip,┴_) components of these fields (which are estimated from the ordering temperature, see Supplementary Note 3) will give rise to a dipolar bias (*ξ*_dip_ = *g*_||_*µ*_B_*B*_dip,||_) and a tunnel splitting (*Δ*_T_ = *g*_┴_*µ*_B_*B*_dip,┴_) of the ground doublet, respectively, so that the total splitting of the ground state is equal to Δ*E* = (*Δ*_T_^2^ + *ξ*_dip_^2^)^½^. Only dysprosium spins for which *ξ*_dip_ < *Δ*_T_ is fulfilled can flip through tunneling^6,50^. However, as a tunneling event at a given site will lead to a redistribution of the dipolar fields throughout the crystal, all spins will eventually enter this tunnel window^6^.

For the unphysical assumption of isotropic dipolar fields we obtain tunnel splittings of *Δ*_T_ = 5.1 × 10^−4^ cm^−1^ and *Δ*_T_ = 1.4 × 10^−4^ cm^−1^, corresponding to tunneling rates of Γ = 6.0 × 10^5^ s^−1^ and Γ = 5.9 × 10^4^ s^−1^ for **1Dy** and **2Dy**, respectively (Supplementary Note 3). The calculated rates overestimate the experimental values by three orders of magnitude. However, considering the pronounced Ising nature of the dysprosium spins at low temperature (experimentally verified by torque magnetometry) and the anisotropic crystal packings (Supplementary Fig. 26) it is physically reasonable to allow the dipolar fields at the dysprosium sites to be anisotropic (*B*_dip,||_ > *B*_dip,┴_) but to retain their overall magnitude. Within such a scenario, the experimentally determined rates of Γ ~ 3 × 10^2^ s^−1^ and Γ ~ 60 s^−1^ are obtained for *Δ*_T_ ~ 1.6 × 10^−5^ cm^−1^ and *Δ*_T_ ~ 6.1 × 10^−6^ cm^−1^ for **1Dy** and **2Dy**, respectively (see Supplementary Note 3).

## Discussion

It appears that the increased symmetry in **2Dy** leads to an approximately threefold reduction of the ground state tunnel splitting as compared to the low-symmetry **1Dy** specimen. We emphasize that the reduction of the tunneling splitting predominantly results from the reduction in the value of the perpendicular component of the ground state **g**-tensor, resulting from the minimization of the off-diagonal *B*_4_^4^ CF parameter. Indeed, due to similar values of *B*_dip,┴_ (Supplementary Note 3), the difference in *g*_┴_ values translates into an equal difference in tunnel splittings.

Interestingly, the fivefold reduction in the tunneling rate is highly similar to the reported threefold to fivefold decrease in *T*_1_^−1^ associated with the removal of dysprosium nuclear spins in dysprosium complexes where the transverse fields are of primarily hyperfine origin^45,53^. The pronounced effect of dilution in the present case indicates that dipolar interactions dominate the effects of nuclear spins. Furthermore, we note that the isothermal susceptibilities (*χ*_T_) are equal to the static values (Supplementary Fig. 23) and that the relaxation processes characterized by the *T*_1_ times given in Fig. 4c account for at least 90 % of the susceptibility of the systems at *T* = 1.9 K (i.e., (*χ*_T_ − *χ*_S_)/*χ*_T_ ≥ 0.9, *χ*_S_ being the adiabatic susceptibility). These observations imply that the dysprosium sites exhibit relaxation on the same timescale (within the distribution width given by *α*) whether bearing nuclear spins or not (naturally occurring dysprosium consists of isotopes with *I* = 0 and *I* = 5/2 in a 0.56:0.44 ratio).

While dipolar fields facilitate tunneling in the studied dysprosium complexes, our results imply that appropriate anisotropy in those fields can serve to decrease the tunneling rate through the combined effect of a reduced tunnel splitting and an increased dipolar bias width (Γ ∝ *Δ*_T_^2^/*σ*_dip_, where *σ*_dip_ is the dipolar bias width, see Supplementary Note 3). This opens the possibility that appropriate tailoring of highly anisotropic crystal packings could lead to relatively concentrated magnetic solids where dipolar fields play a minor role in defining the tunnel-splitting-opening transverse field. This prospect could be of importance to the emerging field of molecular spin qubits^54^.

We note that direct spin–phonon relaxation cannot drive the thermalization of the spins in the tunneling regime (the resulting SLR rates would be unphysically small)^5^. However, the phonon laser effect^55^ can provide an alternate mechanism for achieving thermal equilibrium with the surrounding phonon bath (Supplementary Note 4). The fact that the calculated phonon laser rates (~6 × 10^2^ s^−1^ and ~2 × 10^2^ s^−1^ for **1Dy** and **2Dy**, respectively) are larger than the observed tunneling rates, renders the phonon laser effect a feasible relaxation mechanism in the present case, which in turn suggests that the observed tunneling rates actually reflect the magnitude of the ground state tunnel splitting, rather than phononic insufficiencies in accommodating the thermalization of the electronic spins. Hence the modulation of the SLR rate observed for **1Dy** at low temperatures (cf. Figure 4c) is attributed to the onset of a competition between spin fluctuations caused by tunneling events and the spin-spin correlation length that progressively increases towards *T*_N_^52^. As the ordered phase is entered, the center of the dipolar bias distribution shifts from zero to *ξ*_dip_ ~ *k*_B_*T*_N_ and narrows^5^, hence placing most of the spins outside the tunnel window, giving rise to a decreasing rate. This critical slowing down of the tunneling rate has been observed for a few other molecular nanomagnets^5,51,52^. In accordance with the low *T*_N_ and the significantly smaller tunneling rate in **2Dy**^52^, no discernible modulation of *T*_1_^−1^ is observed in the accessible temperature window. Interestingly, this suggests that if long-range magnetic ordering can be suppressed, the tunneling condition will be very robust. This inhibition is clearly advantageous when seeking control over the timescale of tunneling by means of chemistry.

In conclusion, our work demonstrates how the timescale of quantum tunneling of electronic spins can be controlled and manipulated by chemical means through the targeting of structural conformers of high-symmetry lanthanide complexes. While minimization of tunneling rates through the design of axially symmetric coordination environments has been a long-standing design criterion, our results allow for a quantification of the effect of geometrically imposed electronic linearity, and a comparison with other factors governing the magnitude of ground state tunnel splittings in lanthanide complexes. Furthermore, the engineering of crystal environments with significant dipolar field anisotropy and suppression of long-range magnetic order serve as interesting second generation design criteria worth pursuing in the quest for further control over spin tunneling in the bulk phase of already symmetry-optimized lanthanide complexes. We propose, that if the conformational change can be induced by external stimuli (e.g., electric voltage or current), the studied complex could hold great promise as a building unit in nanoscopic spin-based devices.

## Methods

### Synthetic methods

**1Dy**, **2Dy**, **1Y**, and **2Y** were synthesized according to the published procedure^33^. The solid solution **1Y**_**0**.**95**_**Dy**_**0**.**05**_ was synthesized following the protocol for **1Y**, employing YCl_3_·6H_2_O and DyCl_3_·6H_2_O in a 19:1 molar ratio. The purity of the solid solution was verified using powder X-ray diffraction (Supplementary Fig. 27). The diffraction data were collected at room temperature on a Bruker D8 ADVANCE powder diffractometer operating in a 2*θ*–*θ* configuration using Co Kα radiation (*λ* = 1.7902 Å).

### CF modeling

The CF modeling based on spectroscopic data, molar magnetization, and molar magnetic susceptibility was performed using the MagProp software^56^. In the fitting procedure, the spectroscopic observations were included with their relevant uncertainties, while the molar magnetization and molar magnetic susceptibility data were included in *M*(*BT*^−1^) format. *χ*^2^ was minimized using a tridiagonal decomposition diagonalization algorithm with a 1/d*y*^2^ weighting scheme. In order to allow for an error in the mass of the sample used for magnetization and susceptibility measurements, a model scaling factor was included in the fit. As argued elsewhere^33^, the minimal, common point group symmetry at the dysprosium site in **1Dy** and **2Dy** is *C*_4_. For this reason, the appropriate Hamiltonian of the systems in the Stevens formalism is^34^

3 $$\hat H = \hat H_{{\mathrm{Zeeman}}} + \hat H_{{\mathrm{CF}}} = g_J\mu _{\mathrm{B}}{\bf{B}} \cdot {\hat{\bf J}} + \mathop {\sum}\limits_{k = 2,4,6} {B_k^0} \hat O_k^0 + B_4^{ + 4}\hat O_4^{ + 4} + B_6^{ \pm 4}\hat O_6^{ \pm 4}$$

where, as discussed in the main text, the first term accounts for the Zeeman effect and the latter terms account for CF effects. The definitions of the respective Stevens operators are given elsewhere^35^. If the full CF Hamiltonian is applied, six CF parameters have to be determined. However, it proved impossible to obtain an unambiguous fit if all the off-diagonal terms were included. According to common practice for tetragonal or near-tetragonal systems^5,6,57^, the problem was overcome through the exclusion of the sixth order off-diagonal operators (i.e., those associated with the *B*_6_^±4^ parameters), allowing the *B*_4_^+4^*Ô*_4_^+4^ term (which appears in the main text as *B*_4_^4^*Ô*_4_^4^ for convenience) to be the only one describing the low-symmetry components of the CF.

### Luminescence spectroscopy

Luminescence data were obtained on powdered specimens of **1Dy** and **2Dy** using a HORIBA Jobin Yvon Fluorolog fluorimeter equipped an Oxford Instruments helium flow cryostat, a photomultiplier, and an InGaAs NIR detector.

### Inelastic neutron scattering

INS data were acquired using the direct geometry multi-chopper cold neutron time-of-flight spectrometer TOFTOF^58,59^ at Forschungsneutronenquelle Heinz Maier-Leibnitz FRM II, Garching, Germany and the direct geometry high-flux thermal neutron time-of-flight spectrometer IN4C^60^ at Institut Laue-Langevin (ILL), Grenoble, France. For the measurements on TOFTOF, an aluminum foil bag containing between 1.3 and 1.6 g of polycrystalline sample was rolled into a cylinder and inserted into a hollow, cylindrical aluminum can. For the measurements on IN4C the Al foil bag was inserted in a rectangular cadmium frame, and mounted at an angle of 135° to the incoming neutron beam. At IN4C, a standard ILL Orange cryostat was used for temperature control, while at TOFTOF, a standard top-loading cryostat was used for this purpose. All data were analyzed using the LAMP program package^61^. The data were corrected for detector efficiency using data from a vanadium sample, and the data taken on TOFTOF were corrected for the background associated with the sample holder by performing an empty can measurement. Model INS spectra were simulated using the “ins” program described elsewhere^62^.

### Electron paramagnetic resonance spectroscopy

Powder X-band EPR spectra were acquired at low temperatures (*T* < 15 K) on a Bruker Elexsys E500 spectrometer equipped with a Bruker SUPER-X CW-EPR bridge, a Bruker ER 4116 DM dual mode cavity, and an EIP 538B frequency counter.

### Cantilever torque magnetometry

Single crystals of **2Dy** suitable for cantilever torque magnetometry were prepared using the published method for growing EPR suitable single crystals^33^. A square-shaped crystal of **2Dy** (34 µg) was indexed using an Xcalibur3 single crystal diffractometer equipped with a Sapphire 3 CCD detector. The indexed crystal was then attached to the cantilever (upper plate of a capacitor able to elastically bend) with grease and cooled down to 5 K. Rotations of the cantilever inside a homogenous external magnetic field of the order of Teslas allowed for determination of the magnetic torque (**τ** **=** **M** × **B**) as simply proportional to the difference in capacitance between the capacitor plates.

### Heat capacity

The temperature dependence of the heat capacity *C*(*T*), between 1.8 K and 300 K under zero applied field, was measured on pressed pellets of **1Y** and **2Y** fixed with Apiezon N grease using a Quantum Design Physical Properties Measurement System (PPMS).

### SQUID magnetometry

The magnetization and dc magnetic susceptibility data for **1Dy** and **2Dy** were reported elsewhere^33^. The ac magnetic susceptibility measurements were conducted using a Quantum Design MPMS-XL SQUID magnetometer equipped with a 5 T dc magnet. Data were collected at selected frequencies between 1 and 1488 Hz with an oscillating magnetic field of 0.3 mT with or without an applied dc field up to 500 mT. For all measurements, between 20 and 30 mg of sample was ground into a powder and loaded into a polycarbonate capsule, gently compressed and covered in hexadecane. Fitting of the frequency dependent ac magnetic susceptibility was carried out using the CC-fit program developed by Nicholas F. Chilton^63^.

### mK ac susceptometry

The ac susceptometer was mounted in an Oxford Instruments Kelvinox400 dilution fridge. For each sample, between 20 and 30 mg was ground into a powder and embedded in Stycast in order to cast a cylinder fitting within the susceptometer. The samples were thermalized by 4 copper wires, each 200 µm in diameter. One end of the wires was encapsulated along with the sample in the Stycast and the other end was attached to the weak link of the dilution fridge. The raw frequency dependent data suffered from a frequency dependent background, and the imaginary component had to be corrected for this prior to Cole–Cole fitting of the data. As it was not feasible to carry out a measurement of a blank, the background was instead determined in the following way. Due to the larger magnitude of the real component of the susceptibility relative to the imaginary one, the former response was much less affected by the background. A reasonable fit of *χ´*(*ν*_ac_) to the expression for the in-phase component within the Cole–Cole model^44^ could therefore be obtained. The so-obtained parameters were used to simulate the corresponding out-of-phase component. By subtracting the simulated response from the experimental one, the background could be determined. This procedure was carried out at different temperatures, and the background response was found to be temperature independent. For this reason, a universal background was used for each measured sample. Following the background correction, fitting of the frequency dependent data was carried out using the CC-fit program as described above.

### Data availability

The complete set of experimental data from the IN4C INS experiment can be found in ref.^60^. All other relevant data from this study are available from the authors on request.

## Electronic supplementary material

Supplementary Information (PDF 3644 kb)
