# Supplementary material for: Chemical tunnel-splitting-engineering in a dysprosium-based molecular nanomagnet
Source: Nat Commun. 2018 Mar 29;9:1292. doi: 10.1038/s41467-018-03706-x (PMC5876375; doi:10.1038/s41467-018-03706-x)
Supplement: Supplementary file 1 — Supplementary Information (PDF 3644 kb) [file 41467_2018_3706_MOESM1_ESM.pdf]

Chemical tunnel-splitting-engineering in a dysprosium-based molecular nanomagnet

Mikkel A. Sørensen *et al*.

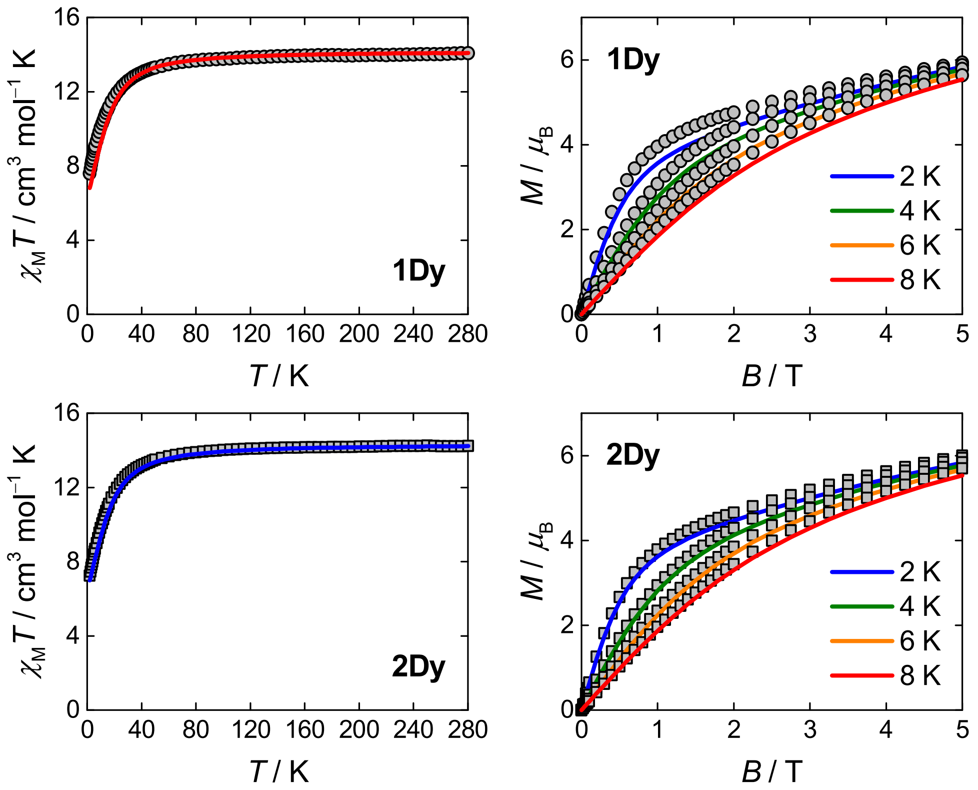

Supplementary Figure 1 $\chi_M T$ products and molar magnetization isotherms for **1Dy** and **2Dy**. The solid lines represent the global best fit as described in the main text. The experimental data were originally reported elsewhere[1]. The applied scaling factors (in order to account for error in the mass of the sample used) are 1.002 and 1.012 for **1Dy** and **2Dy**, respectively.

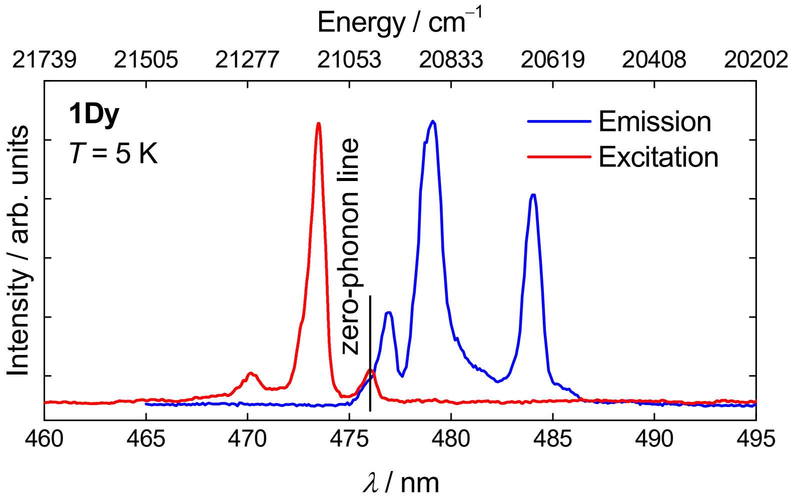

Supplementary Figure 2 $^4F_{9/2} \rightarrow {}^6H_{15/2}$ luminescence spectrum (λ_{ex} = 386 nm) overlaid with the excitation spectrum (λ_{em} = 575 nm) for **1Dy** recorded at *T* = 5 K. The shortest wavelength peak in the emission spectrum coincides with the longest wavelength peak in the excitation spectrum, identifying it as the zero-phonon line (*i.e.* the emission/excitation transition involving the lowest lying substate of the $^6H_{15/2}$ multiplet).

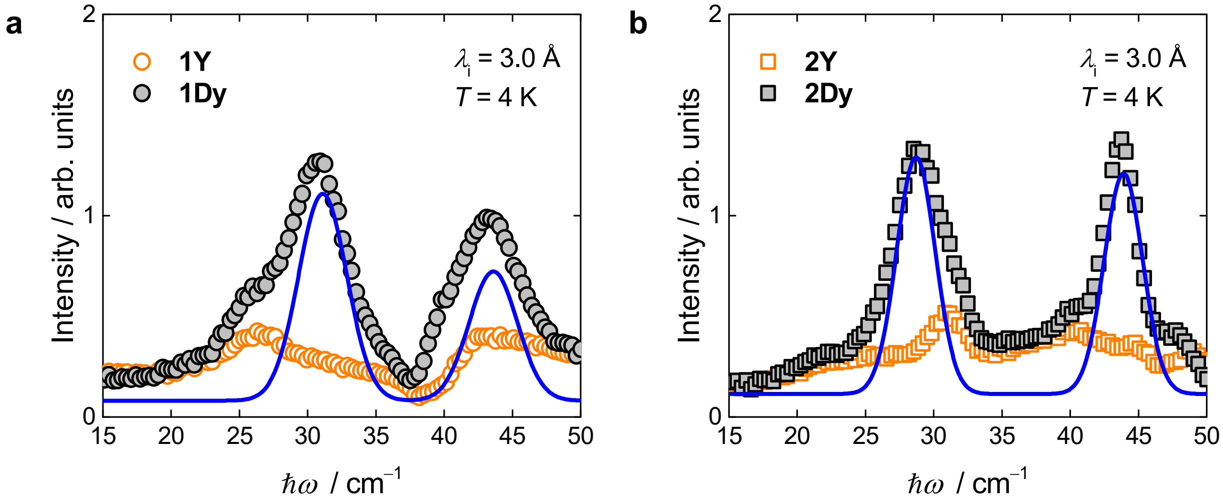

Supplementary Figure 3 INS spectra for **1Dy** and **1Y** (**a**) and **2Dy** and **2Y** (**b**) at T = 4 K and λ_i = 3.0 Å. The data were integrated over 0.95 Å^{-1} ≤ Q ≤ 1.8 Å^{-1}. The blue lines give the INS spectra simulated using the CF parameters described in the main text and the relevant experimental parameters. In both figures, the errors are less than the size of the symbols. The data were recorded on TOFTOF.

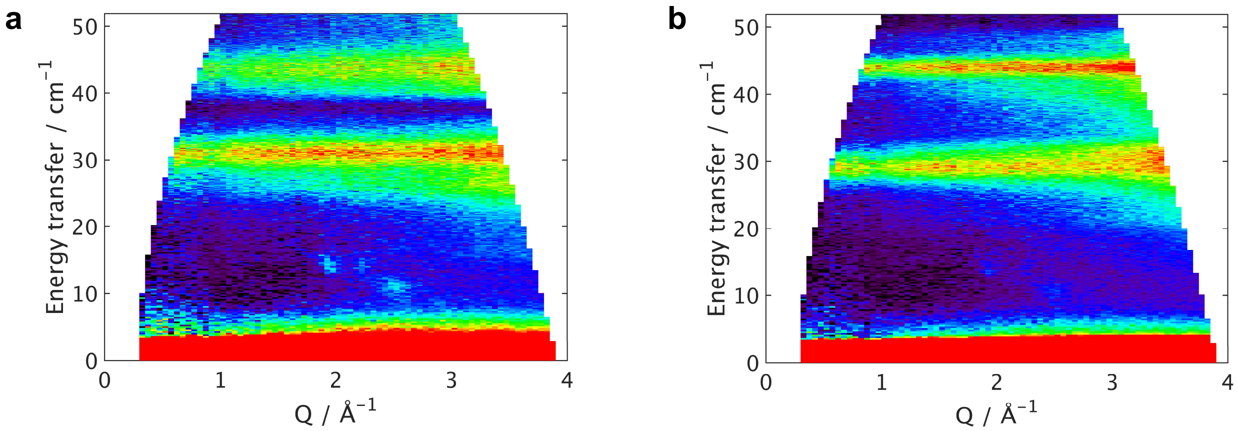

Supplementary Figure 4 $S(Q,\omega)$ plots for **1Dy** (**a**) and **2Dy** (**b**) at T = 4 K and λ_i = 3.0 Å. Red (dark blue) denotes maximum (minimum) scattering intensity. Note that the difference between **1Dy** and **2Dy** in the relative intensity of the two CF excitations, discussed in the main text, is immediately apparent in the $S(Q,\omega)$ maps. The data were recorded on TOFTOF.

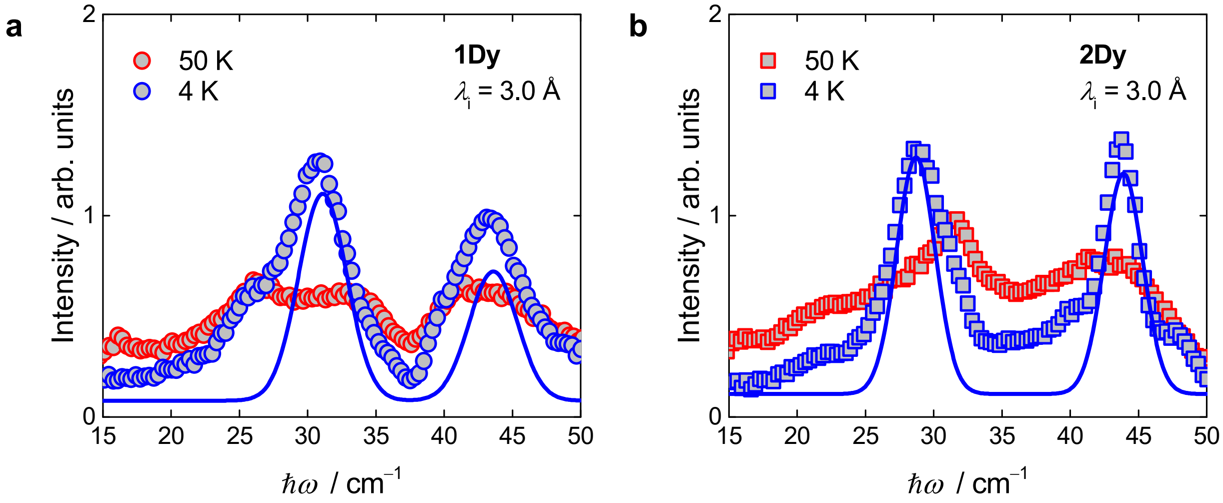

Supplementary Figure 5 INS spectra for **1Dy** (**a**) and **2Dy** (**b**) at T = 4 K (blue) and T = 50 K (red) recorded with an incident neutron wavelength of λ_i = 3.0 Å. The data were integrated over 0.95 Å^{-1} ≤ Q ≤ 1.8 Å^{-1}. The blue lines give the INS spectra simulated using the CF parameters described in the main text and the relevant experimental parameters at T = 4 K. In both figures, the errors are less than the size of the symbols. The data were recorded on TOFTOF.

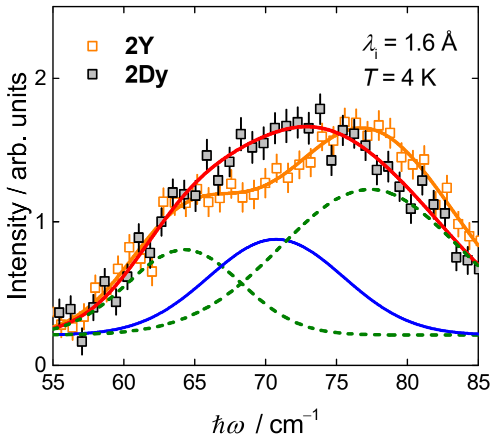

Supplementary Figure 6 Selected part of the INS spectra for **2Dy** (grey squares) and **2Y** (orange squares) at T = 4 K recorded with an incident neutron wavelength of λ_i = 1.6 Å. The data were integrated over 1.2 Å^{-1} ≤ Q ≤ 2.5 Å^{-1}. The spectrum of **2Y** can be well accounted for by a sum of two Gaussians (orange line) in the energy window of interest. As **2Dy** and **2Y** are isostructural, the spectral difference is assigned to the presence of a weak CF excitation in the spectrum of **2Dy**. At λ_i = 1.6 Å, the Gaussian linewidths (full width at half maximum) of the lower-lying CF excitations (the ones from the ground doublet to the first and second excited doublets) were determined to be ~11.2 cm^{-1} (not shown). The spectrum of **2Dy** in the energy transfers between 55 and 85 cm^{-1} was consequently described by a sum of three Gaussians; the two from the deconvolution of the **2Y** spectrum, and a third with a linewidth of 11.2 cm^{-1}. In the fit, the linewidths of the Gaussians accounting for the two phonon modes were fixed to the values determined for **2Y**, but their areas and positions were allowed to vary freely. The best fit is given by the red line, and the individual Gaussians are given as green dashed lines (phonons) or a solid blue line (CF excitation), respectively. The best fit affords a position of the CF excitation of 71(3) cm^{-1}. This value agrees reasonably well with the separation between the ground and third excited doublet of 67 cm^{-1} determined from the CF modelling. As discussed in the main text, this excitation is observable by INS due to the 25 % |±9/2⟩ contribution to the wavefunction of the third excited doublet. The data were recorded on TOFTOF.

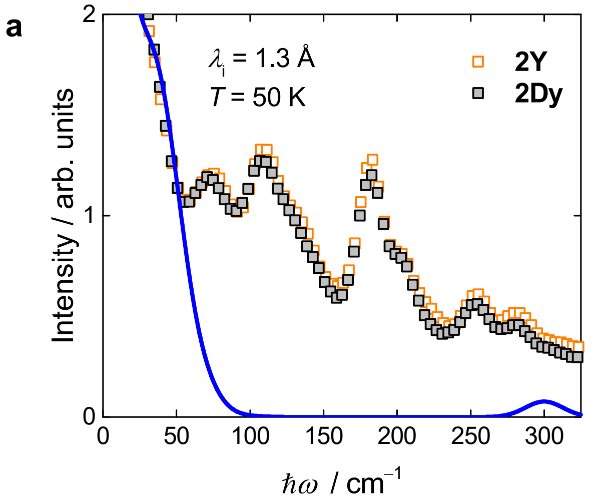

Supplementary Figure 7 INS spectra for **2Dy** (grey squares) and **2Y** (orange squares) at T = 50 K and λ_i = 1.3 Å (**a**) and at T = 20 K and λ_i = 0.84 Å (**b**). The data were integrated over 3 Å^{-1} ≤ Q ≤ 5 Å^{-1} and 2.6 Å^{-1} ≤ Q ≤ 4.6 Å^{-1}, respectively. The blue lines give the INS spectra simulated using the CF parameters described in the main text and the relevant experimental parameters. The peak in the simulated spectrum corresponds to INS allowed CF excitation from the first excited doublet, |±13/2⟩, to the highest lying one, |±15/2⟩. The weak intensity associated with the "hot" nature (*i.e.* originating from an excited state) of the excitation combined with the loss of the relevant low Q-region, and the significant background associated with the incoherent scattering of the hydrogen nuclei contained in the sample, account for the lack of success in observing the excitation. The difficulties associated with observing CF excitations at very high energy transfers for hydrogen containing molecule-based materials have been reported elsewhere[2]. In both figures, the errors are less than the size of the symbols. The data were recorded on IN4C.

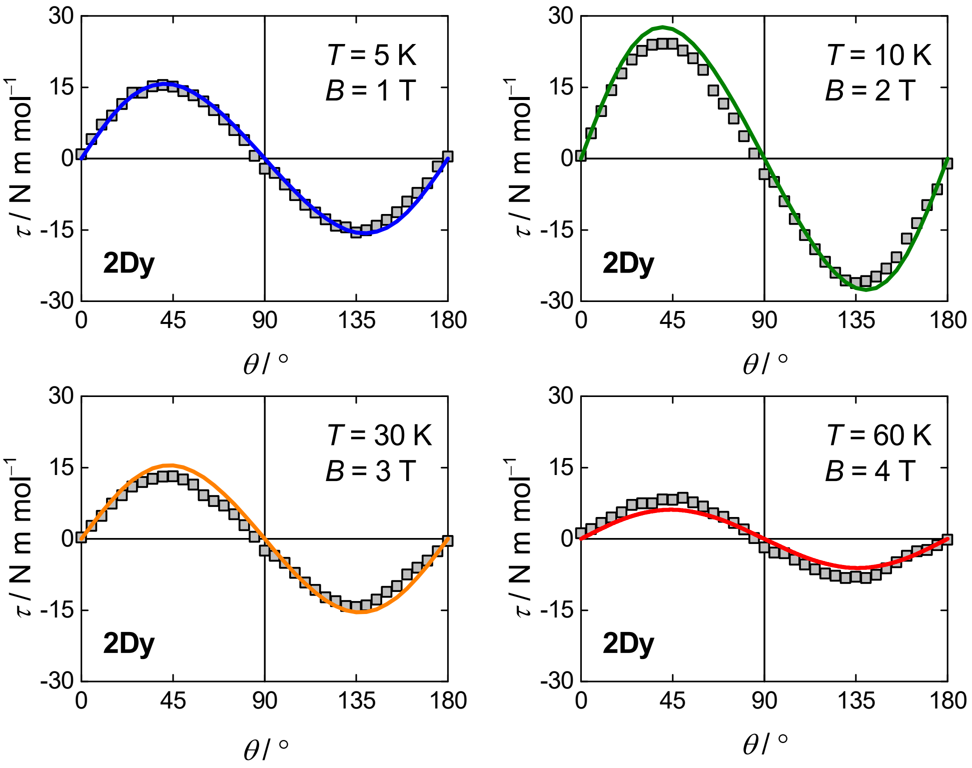

Supplementary Figure 8 Molar magnetic torque of a single crystal of **2Dy** recorded at the indicated temperatures and applied fields. The angle *θ* denotes the angle (increasing anticlockwise) between the *ab* plane and the applied magnetic field (see Supplementary Fig. 9). The solid lines are simulations based on the CF parameters reported in the main text. The upper left panel is equivalent to Fig. 2f of the main text and is included here as reference.

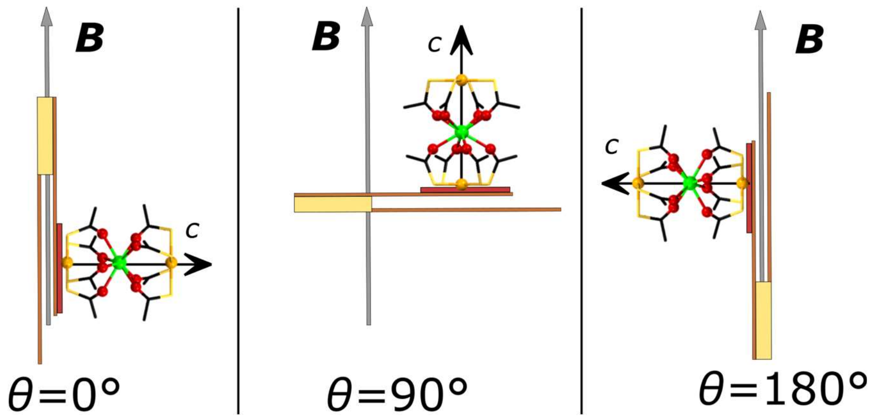

Supplementary Figure 9 Schematics of the experimental setup for the cantilever torque magnetometry experiment along with the definition of the θ angle. Due to the macroscopic shape of the crystal (thin square-shaped plate) it was only possible to perform the rotation from the ab plane to the *c* axis. A simulation of the in-plane (the *ab* plane that is) rotation (Supplementary Fig. 10) demonstrates that its intensity is at least three orders of magnitude lower than the rotation experimentally performed. This justifies our choice to fix the crystallographic *abc* reference frame to be coincident with the magnetic *xyz* reference frame.

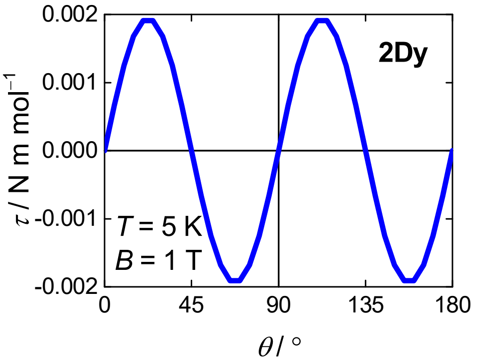

Supplementary Figure 10 Simulation of the in-plane rotation for **2Dy**. Note that the signal is at least three orders of magnitude smaller than that observed for the rotation performed experimentally (*i.e.* the rotation from the *ab* plane to the *c* axis, see Supplementary Fig. 8).

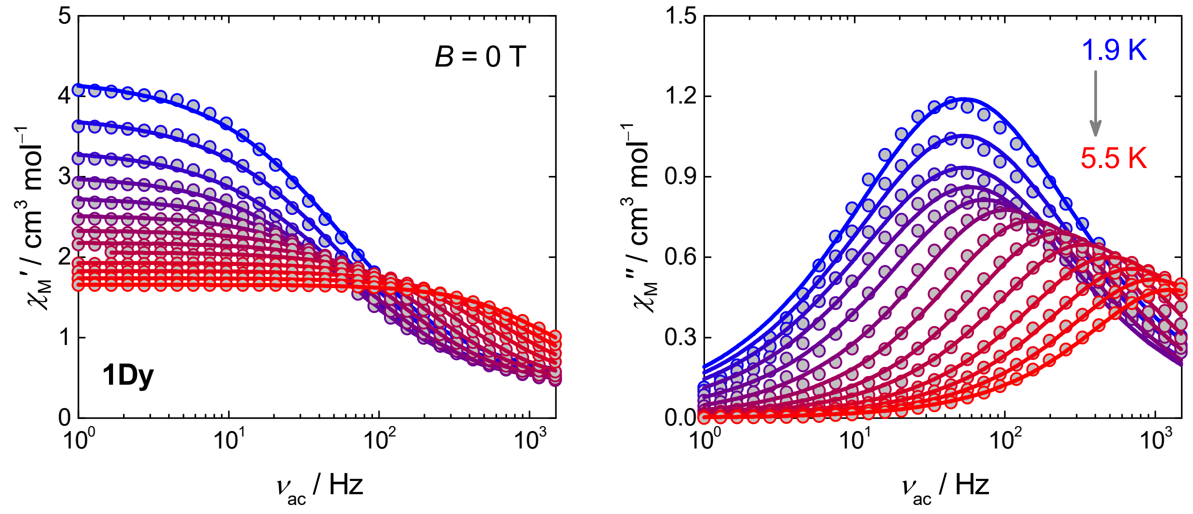

Supplementary Figure 11 Frequency dependence of the ac molar magnetic susceptibility for **1Dy** in zero applied dc field measured at selected temperatures between 1.9 K and 5.5 K. Solid lines are best fits to a Cole-Cole function.

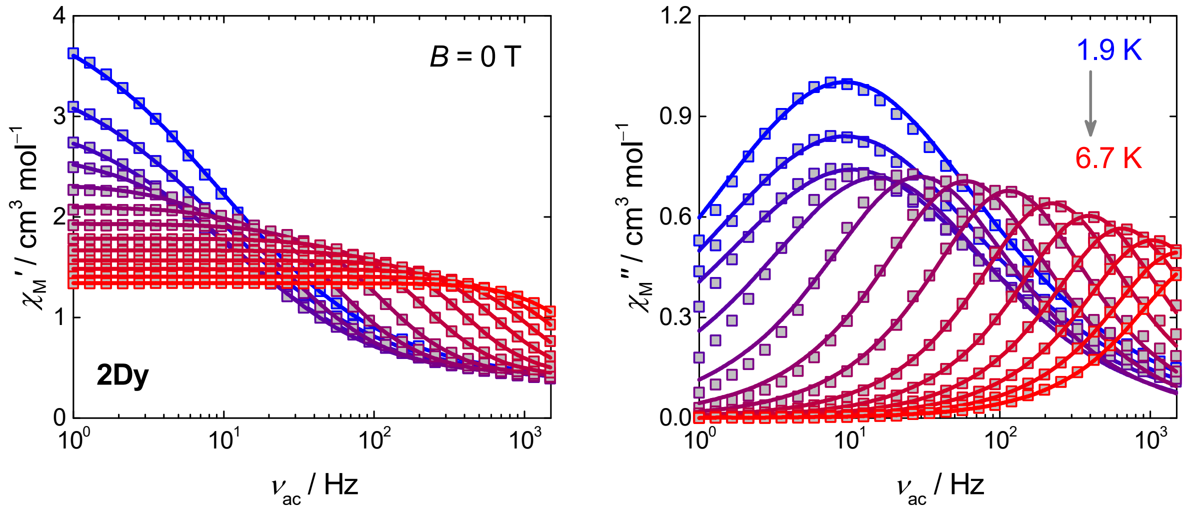

Supplementary Figure 12 Frequency dependence of the ac molar magnetic susceptibility for **2Dy** in zero applied dc field measured at selected temperatures between 1.9 K and 6.7 K. Solid lines are best fits to a Cole-Cole function.

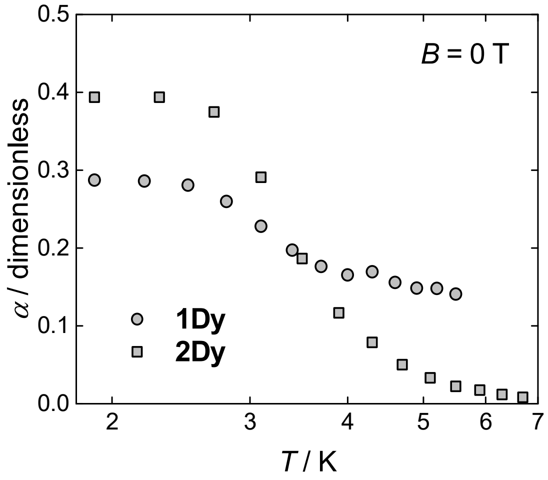

Supplementary Figure 13 Temperature dependence of the relaxation time distribution parameter for **1Dy** and **2Dy** in zero applied dc field. The data were extracted from Cole-Cole fitting of the data in Supplementary Figs 11 and 12.

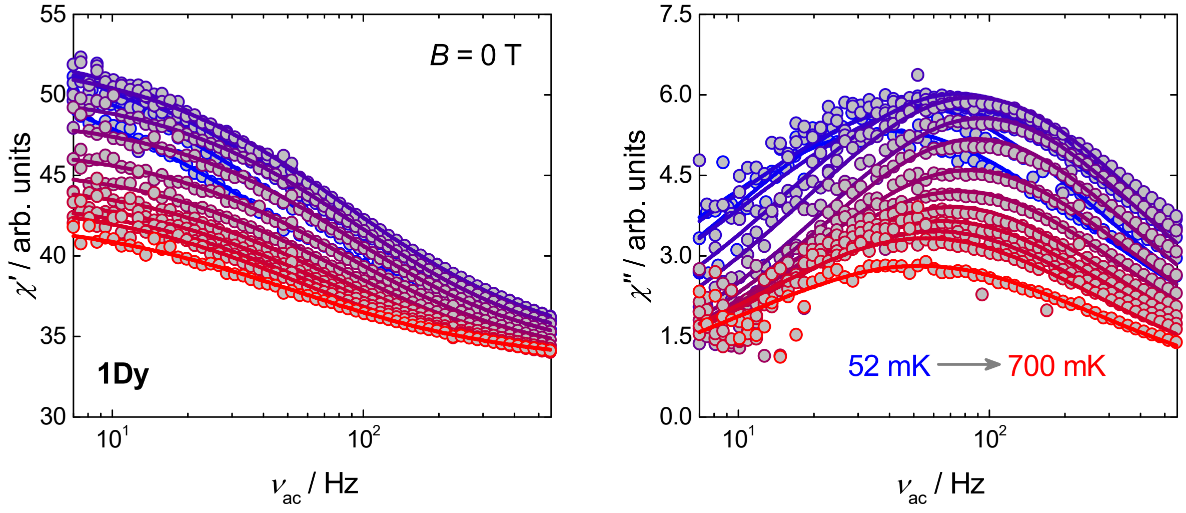

Supplementary Figure 14 Frequency dependence of the ac magnetic susceptibility for **1Dy** in zero applied dc field measured at selected temperatures between 52 mK and 700 mK. Solid lines are best fits to a Cole-Cole function.

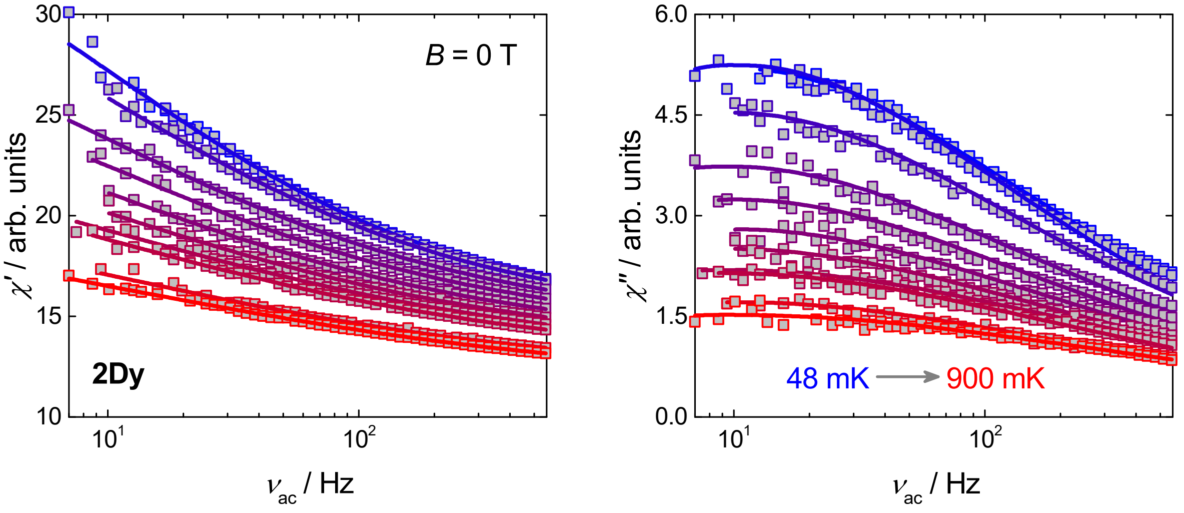

Supplementary Figure 15 Frequency dependence of the ac magnetic susceptibility for **2Dy** in zero applied dc field measured at selected temperatures between 48 mK and 900 mK. Solid lines are best fits to a Cole-Cole function.

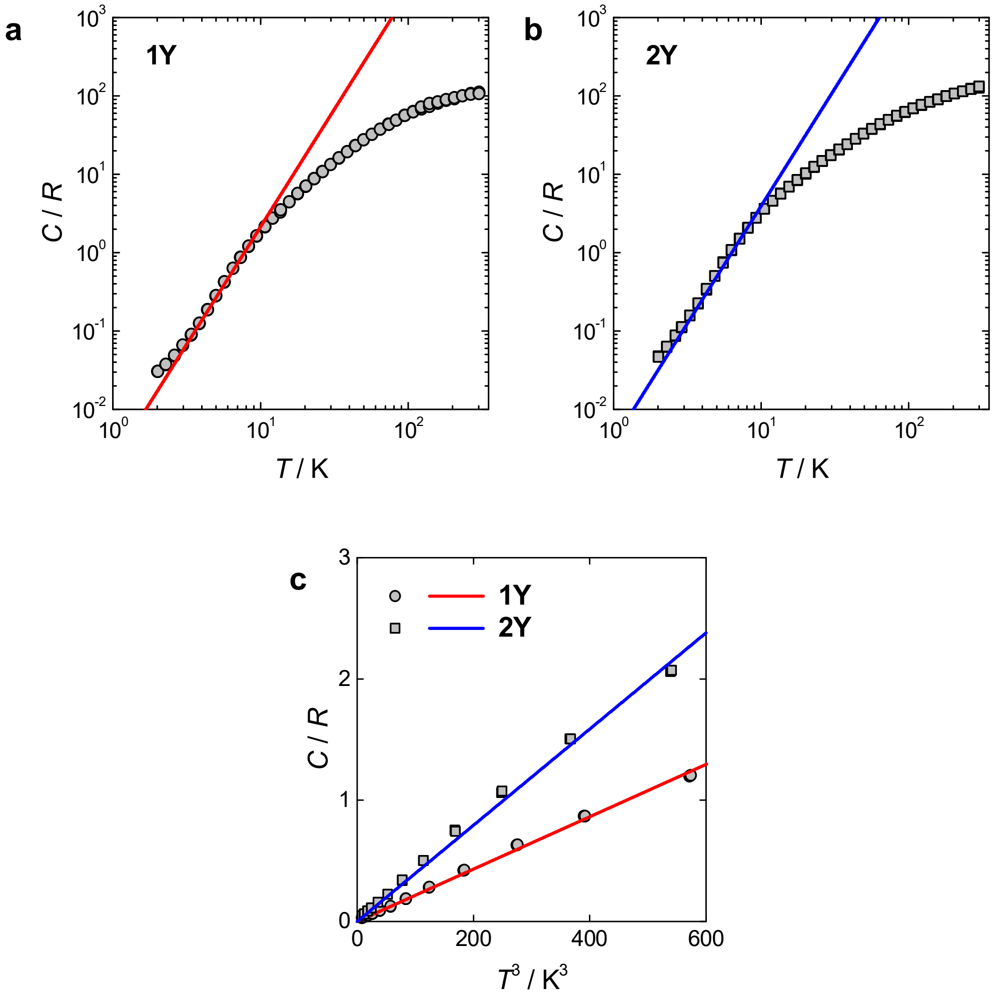

Supplementary Figure 16 Log-log plot of the temperature dependence of the molar specific heat for **1Y** (**a**) and **2Y** (**b**). Within the Debye model, the lattice contribution to the molar specific heat (C/R, R being the gas constant) is given by[3] $C/R = (12\pi^4 r/5)(T/\theta_D)^3$, where r is the number of atoms per formula unit, T is the temperature, and θ_D is the Debye temperature. As the expression only applies for $T << \theta_D$, a fit of the molar specific heat was only carried out for $T < 9$ K. The so-obtained Debye temperatures are 212(1) K and 183(1) K for **1Y** and **2Y**, respectively. The best fits are given as solid lines. A plot of C/R vs. T^3 highlights the Debye contribution to the specific heat at low temperatures (**c**). Based on their isostructural nature[1] and the strong similarities in the phonon part of the INS spectra of the yttrium and dysprosium derivatives (see Supplementary Figs 3, 6-7), the Debye temperatures for **1Y** and **2Y**, determined above, should be reasonably applicable to the respective dysprosium derivatives. Within the Debye model, the mean phonon velocity, v_m, in a given lattice can be calculated as[3] $v_m = (\theta_D k_B/h)(3N_A\rho/4\pi M_w)^{-1/3}$, where k_B is the Boltzmann constant, h is the Planck constant, N_A is Avogadro's constant, ρ is the crystal density, and M_w is the molar weight. From the Debye temperatures determined above, and the crystal densities determined from the X-ray single crystal structures reported elsewhere[1], values of $v_m = 6.98(3)\cdot10^3$ m s^{-1} and $v_m = 6.55(3)\cdot10^3$ m s^{-1} are obtained for **1Dy** and **2Dy**, respectively. In order to correctly determine the dynamic crystal potential matrix element in the Orbach analysis of the spin-lattice relaxation rates of **1Y$_{0.95}$Dy$_{0.05}$** (Supplementary Fig. 22), the mean phonon velocity of **1Y**, also needs to be calculated. Using the appropriate molar weight and crystal density, a mean phonon velocity of $v_m = 6.98(3)\cdot10^3$ m s^{-1} is found for **1Y**, the same as for **1Dy**.

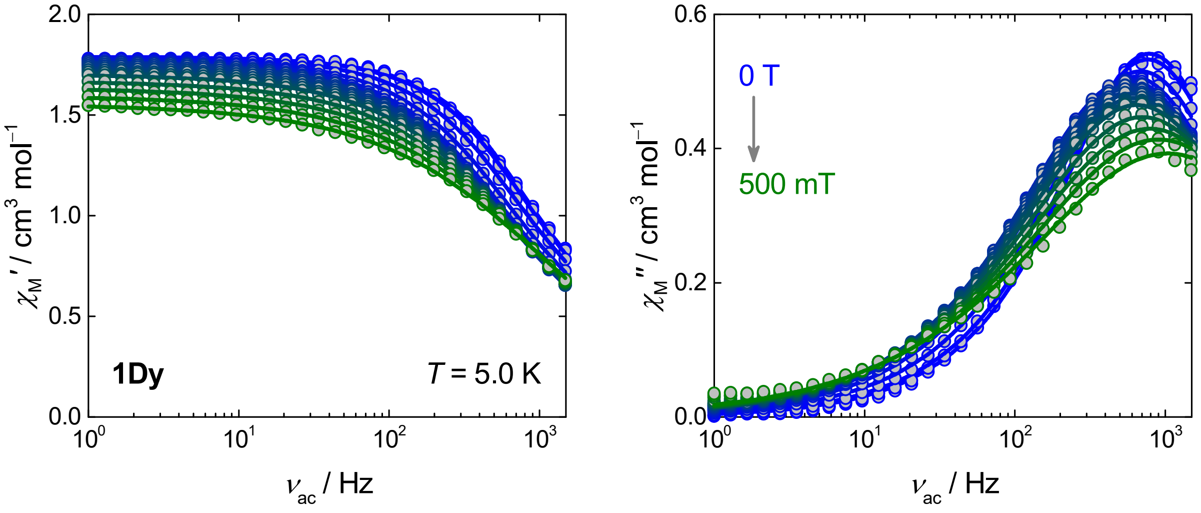

Supplementary Figure 17 Frequency dependence of the ac molar magnetic susceptibility for **1Dy** at T = 5.0 K measured in selected applied dc fields between 0 T and 500 mT. Solid lines are best fits to a Cole-Cole function.

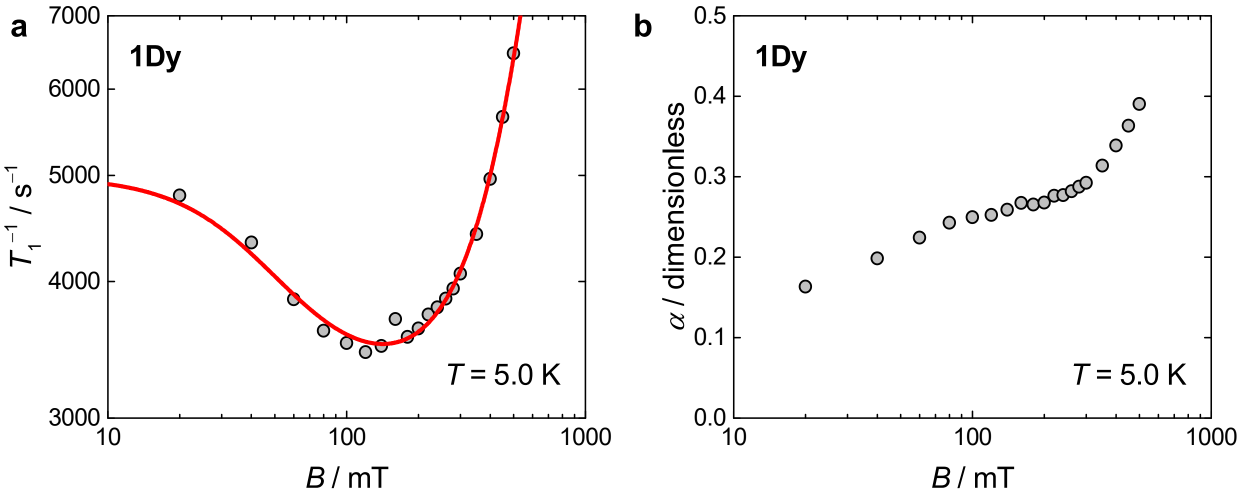

Supplementary Figure 18 Field dependence of the spin-lattice relaxation rate (**a**) and the distribution parameter (**b**) for **1Dy** at T = 5.0 K. The data were extracted from Cole-Cole fitting of the data in Supplementary Fig. 17. The field dependence of the spin-lattice relaxation rate can in the general case be described by the expression[4] $T_1^{-1}(B,T) = B_1/(1+B_2B^2) + ATB^n + k(T)$, where the first term accounts for tunneling, the second for direct relaxation between the two components of the ground doublet, and the last constant term absorbs any field independent relaxation (Orbach relaxation in this case). The best fit of the $T_1^{-1}(B)$ data for **1Dy** (**a**) to this expression with T = 5.0 K yields parameter values of $B_1 = 1.77(8)\cdot10^3$ s^{-1}, $B_2 = 4.6(8)\cdot10^{-6}$ mT^{-2}, $A = 6.1(2)\cdot10^{-5}$ s^{-1} K^{-1} mT$^{-2.6}$, n = 2.6 (fixed), and $k(T) = 3.22(4)\cdot10^3$ s^{-1}. For the standard direct process n = 4, but such a field dependence does not describe the high field data well. The reduced value of the exponent indicates that the direct relaxation process is phonon-bottlenecked, which is supported by the distinct increase in the value of α at high fields (**b**)[5]. The field dependence of the spin-lattice relaxation rate for **1Dy** demonstrates that at zero field and temperatures as high as T = 5.0 K, tunneling within the ground state still influences the measured rates.

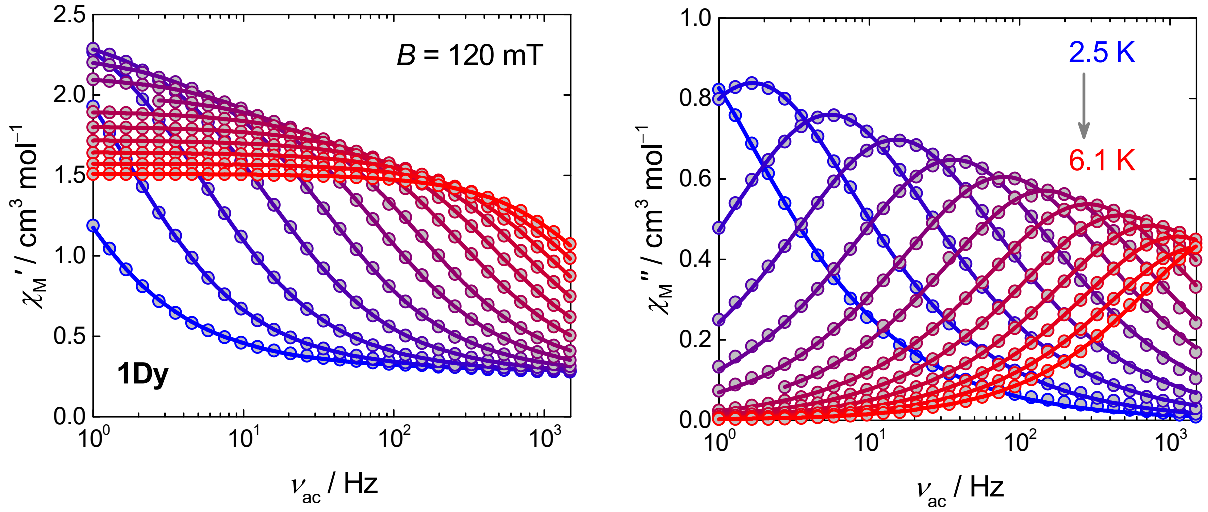

Supplementary Figure 19 Frequency dependence of the ac molar magnetic susceptibility for **1Dy** in an applied field of *B* = 120 mT measured at selected temperatures between 2.5 K and 6.1 K. Solid lines are best fits to a Cole-Cole function.

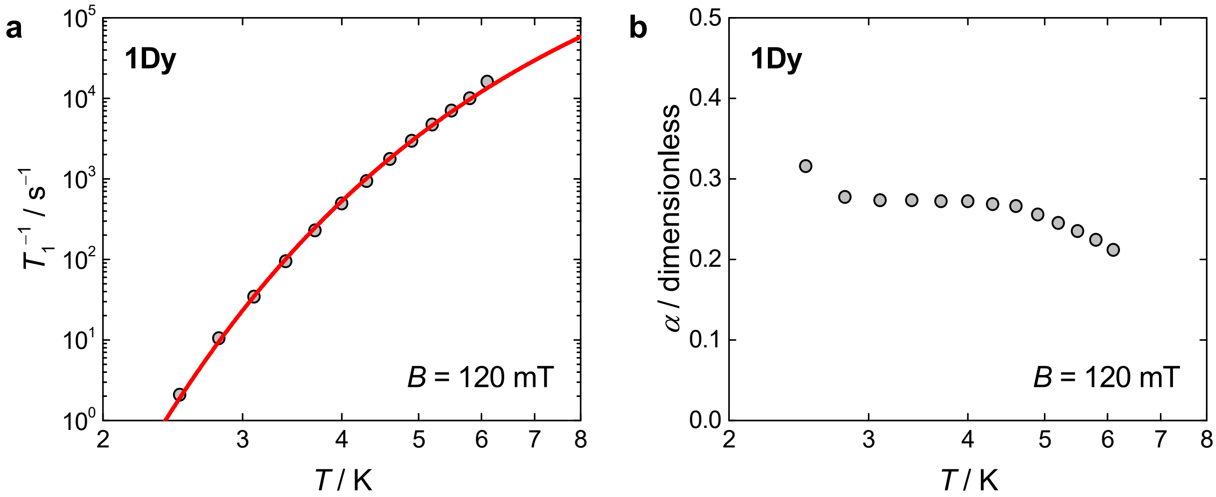

Supplementary Figure 20 Temperature dependence of the spin-lattice relaxation rate (**a**) and the distribution parameter (**b**) for **1Dy** in an applied field of B = 120 mT. The data were extracted from Cole-Cole fitting of the data in Supplementary Fig. 19. The temperature dependence of the spin-lattice relaxation rate can be modelled using the expression $T_1^{-1}(T) = (3M_O^2\Delta^3)/(\pi\hbar^4\rho v^5[\exp(\Delta/k_B T)-1])$, corresponding to pure Orbach relaxation (the expression is identical to the second term in Equation (2) of the main text). By applying the crystal density obtained from the X-ray single crystal structures reported elsewhere[1], and setting $v = v_m$ (obtained from specific heat) for **1Dy**, values of Δ = 26.1(2) cm^{-1} and M_O^2 = 5.6(4)·10^2 cm^{-2} are obtained.

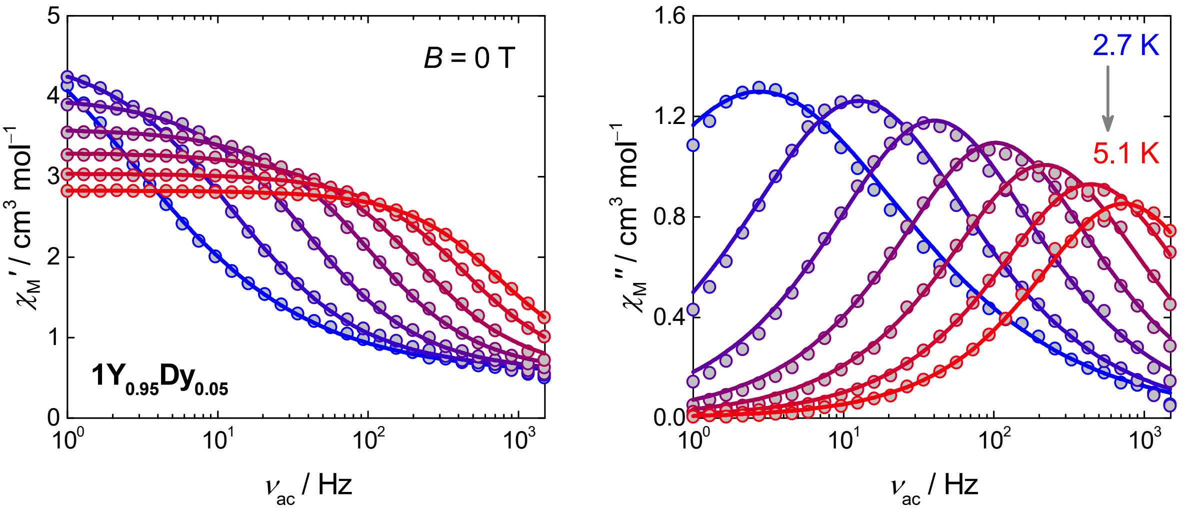

Supplementary Figure 21 Frequency dependence of the ac molar magnetic susceptibility for **$1Y_{0.95}Dy_{0.05}$**, in zero applied field, measured at selected temperatures between 2.5 K and 6.1 K. Solid lines are best fits to a Cole-Cole function.

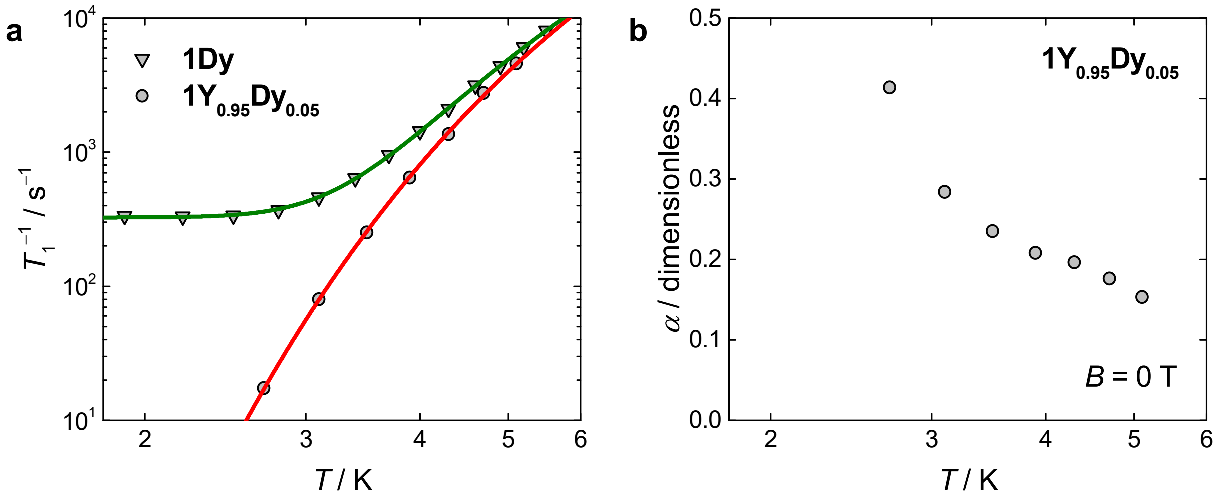

Supplementary Figure 22 Temperature dependence of the spin-lattice relaxation rate (**a**, circles) and the distribution parameter (**b**) for **1Y$_{0.95}$Dy$_{0.05}$** in zero applied field. The data were extracted from Cole-Cole fitting of the data in Supplementary Fig. 21. The solid red line is a best fit to the expression $T_1^{-1}(T) = (3M_O^2\Delta^3)/(\pi\hbar^4\rho v^5[\exp(\Delta/k_BT)-1])$, corresponding to pure Orbach relaxation. The best-fit parameter values are Δ = 22.2(2) cm^{-1} and M_O^2 = 3.2(2)·10^2 cm^{-2}. The triangles give the spin-lattice relaxation rate for the concentrated specimen **1Dy**, and the green line is the best fit of that data (equivalent to the best fit given in Fig. 4c of the main text).

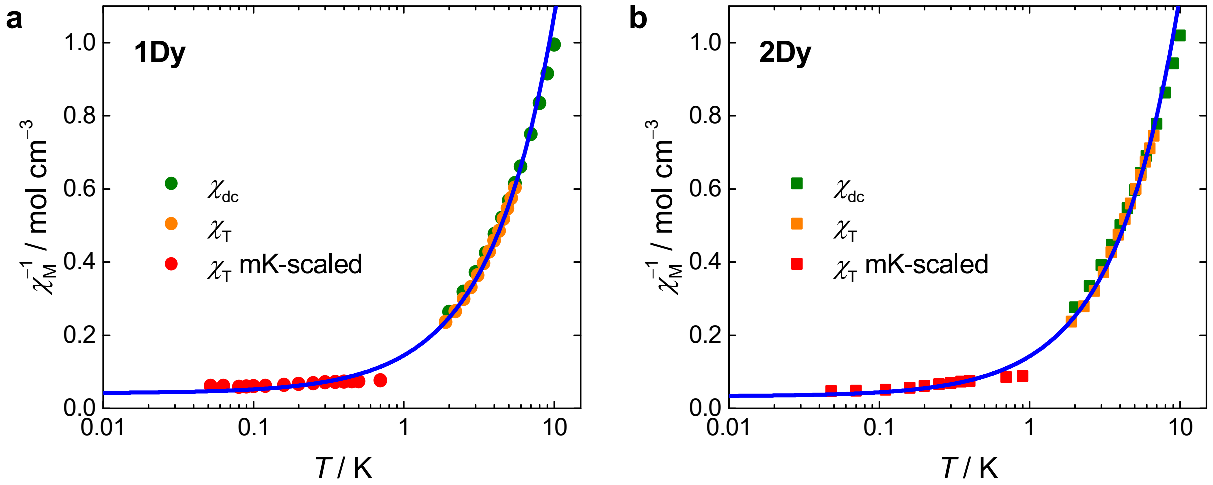

Supplementary Figure 23 Temperature dependence of the inverse isothermal susceptibility (orange circles/squares, determined from the Cole-Cole fitting of magnetic ac susceptibilities) and the inverse dc susceptibility (green circles/squares) for **1Dy** (**a**) and **2Dy** (**b**). As quantification of the mK susceptibility was not possible with the utilized setup, the mK data have been scaled to the high temperature magnetic susceptibility data. The scaled data are given as red circles/squares. The abscissa is logarithmic in order to emphasize the mK data. The blue lines are best fits to a Curie-Weiss law, with Weiss constants of θ = –0.40(3) K, and θ = –0.30(8) K for **1Dy** and **2Dy**, respectively.

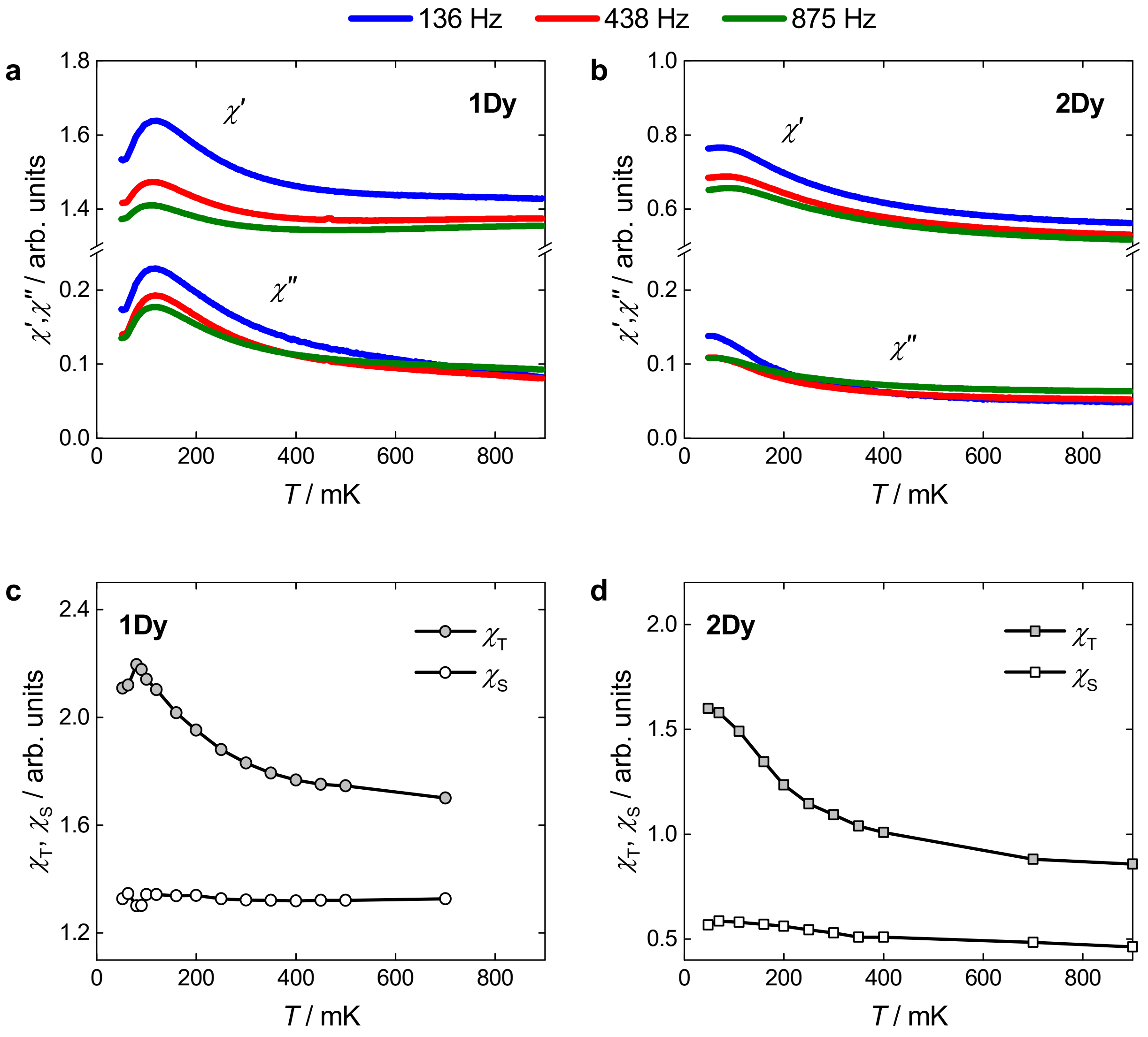

Supplementary Figure 24 Temperature dependence of the ac magnetic susceptibility in the mK regime, at the indicated frequencies, for **1Dy** (**a**) and **2Dy** (**b**). Temperature dependence of the isothermal (grey symbols) and adiabatic (open symbols) susceptibilities for **1Dy** (**c**) and **2Dy** (**d**) obtained from the Cole-Cole fitting of the frequency dependent data in Supplementary Figs 14 and 15. The appearance of maxima in the $\chi'(T)$ curves (**a** and **b**) signals the onset of long-range magnetic order. The setup used for the mK susceptibility measurements did not allow for a quantification of the susceptibility, thereby impeding an identification of the nature of the magnetic order through the comparison with a demagnetization factor. However, a Curie-Weiss analysis of the isothermal susceptibilities (Supplementary Fig. 23) demonstrates the overall interactions between dysprosium ions to be antiferromagnetic in nature. The maxima in the $\chi''(T)$ curves suggest the presence of an uncompensated moment in the ordered phase, something that is incompatible with antiferromagnetic ordering given the symmetry of the crystals. However, as the spin dynamics are on the timescale of the experiment, these are in fact the origin of the maxima in the $\chi''(T)$ curves. As shown in panels **c** and **d**, the temperature dependence the isothermal susceptibility, obtained from Cole-Cole fitting of the frequency dependent data, mimics the temperature dependence of the $\chi'(T)$ curves while the adiabatic susceptibility is essentially temperature independent. As the dynamics are on the timescale of the experiment, the product $2\pi\nu_{ac}\tau$ is not too far from unity, giving rise to a non-zero $\chi''(T)$ response following Supplementary Equation (3) (see Supplementary Note 1). As τ and α (**1Dy**: $0.25 \leq \alpha \leq 0.39$, **2Dy**: $0.49 \leq \alpha \leq 0.62$) only show limited variation in the mK regime, the temperature dependence of the imaginary response must be dominated by that of the isothermal susceptibility. In this way, a maximum appears in the $\chi''(T)$ curves for **1Dy**, while an onset of a peak is observed for **2Dy**. Based on these considerations, we assign the appearance of maxima in the $\chi'(T)$ curves to the onset of long-range antiferromagnetic ordering.

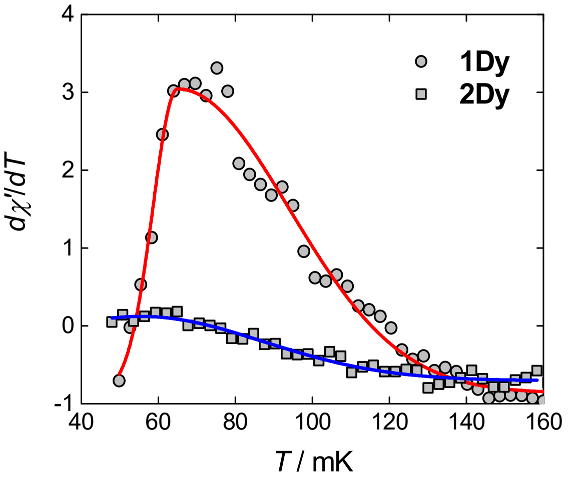

Supplementary Figure 25 $d\chi'/dT$ at v_{ac} = 136 Hz for **1Dy** (circles) and **2Dy** (squares). The maxima were estimated from best-fits to a Bigaussian (red line), and a Gaussian (blue line), respectively. Critical temperatures for long-range antiferromagnetic ordering of T_N = 65 mK and T_N ~ 50 mK were obtained for **1Dy** and **2Dy**, respectively. As no clear maximum is observed for **2Dy**, the estimated value represents an upper limit for T_N.

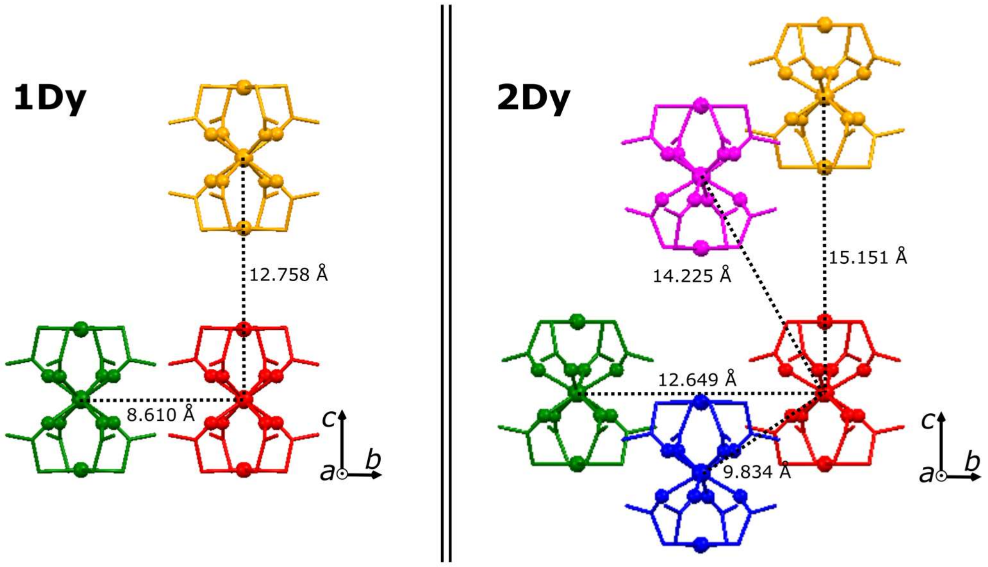

Supplementary Figure 26 Crystal packings of **1Dy** and **2Dy** along *a*, with indications of relevant intermolecular Dy···Dy distances. Note that all molecules are oriented with their fourfold axis (experimentally verified to be the easy axis of magnetization from torque magnetometry) along the crystallographic *c* axis. As the ground state **g**-tensors are highly anisotropic with $g_{||} >> g_{\perp}$, the dipolar field arising from the neighboring dysprosium spins is expected to fulfill the condition $B_{dip,||} > B_{dip,\perp}$ at any given Dy site.

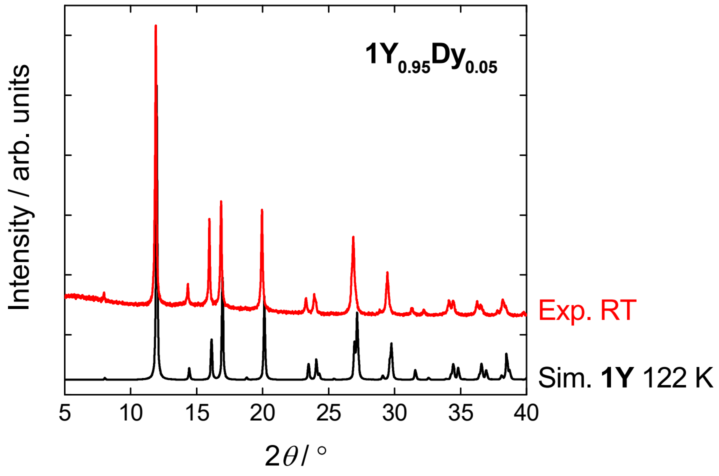

Supplementary Figure 27 Room temperature PXRD pattern of $\mathbf{1Y_{0.95}Dy_{0.05}}$ (recorded using Co Kα radiation, λ = 1.7902 Å) compared to the pattern of **1Y** simulated from the structure recorded at T = 122(1) K (CCDC 1526323)[1].

Supplementary Table 1 $|m_J\rangle$ contributions to the wavefunctions of the crystal field states within the ground Russell-Saunders multiplet for **1Dy**. Only contributions of 1 % or larger have been included. The third column gives twice the expectation value of the projection of the total angular momentum operator along *z* ($2\langle J_z\rangle$), calculated using the relation $\langle J_z\rangle g_J\mu_B = (-\partial E/\partial B)\mu_B$, where g_J is the Landé *g*-value (g_J = 4/3 for Dy^{3+}) and $-\partial E/\partial B$ is computed as part of the MagProp output. In the case of a diagonal CF Hamiltonian $\langle J_z\rangle = m_J$. In order to make the dominantly contributing $|m_J\rangle$ state easily identifiable, the value of $2\langle J_z\rangle$ for each eigenstate is reported below.

Energy / cm^{-1}	Wavefunction	$2\langle J_z\rangle$
0	97 % $|\pm11/2\rangle$, 2 % $|\pm3/2\rangle$	$\pm$10.7579
31.10	87 % $|\pm9/2\rangle$, 11 % $|\pm1/2\rangle$, 2 % $|\mp7/2\rangle$,	$\pm$7.7219
43.60	94 % $|\pm13/2\rangle$, 5 % $|\pm5/2\rangle$	$\pm$12.4268
64.63	70 % $|\pm7/2\rangle$, 21 % $|\mp1/2\rangle$, 9 % $|\mp9/2\rangle$	$\pm$3.8484
75.19	5 % $|\pm13/2\rangle$, 47 % $|\pm5/2\rangle$, 46 % $|\mp3/2\rangle$, 2 % $|\mp11/2\rangle$	$\pm$1.4051
108.0	28 % $|\pm7/2\rangle$, 68 % $|\mp1/2\rangle$, 4 % $|\mp9/2\rangle$	$\pm$0.8843
117.8	48 % $|\pm5/2\rangle$, 50 % $|\mp3/2\rangle$	$\pm$0.9159
326.0	100 % $|\pm15/2\rangle$	$\pm$14.9592

Supplementary Table 2 $|m_J\rangle$ contributions to the wavefunctions of the crystal field states within the ground Russell-Saunders multiplet for **2Dy**. Only contributions of 1 % or larger have been included. The third column gives twice the expectation value of the projection of the total angular momentum operator along *z* ($2\langle J_z\rangle$), calculated using the relation $\langle J_z\rangle g_J\mu_B = (-\partial E/\partial B)\mu_B$, where g_J is the Landé *g*-value ($g_J = 4/3$ for Dy^{3+}) and $-\partial E/\partial B$ is computed as part of the MagProp output. In the case of a diagonal CF Hamiltonian $\langle J_z\rangle = m_J$. In order to make the dominantly contributing $|m_J\rangle$ state easily identifiable, the value of $2\langle J_z\rangle$ for each eigenstate is reported below.

Energy / cm^{-1}	Wavefunction	$2\langle J_z\rangle$
0	99 % $|\pm11/2\rangle$, 1 % $|\pm3/2\rangle$	±10.8729
28.70	99 % $|\pm13/2\rangle$	±12.8884
43.90	74 % $|\pm9/2\rangle$, 24 % $|\pm1/2\rangle$, 2 % $|\mp7/2\rangle$	±6.7598
67.02	25 % $|\pm9/2\rangle$, 56 % $|\pm1/2\rangle$, 19 % $|\mp7/2\rangle$	±1.4609
74.17	1 % $|\pm11/2\rangle$, 72 % $|\pm3/2\rangle$, 27 % $|\mp5/2\rangle$	±0.8648
95.60	79 % $|\pm7/2\rangle$, 20 % $|\mp1/2\rangle$, 1 % $|\mp9/2\rangle$	±5.2296
104.7	72 % $|\pm5/2\rangle$, 27 % $|\mp3/2\rangle$	±2.8392
328.6	100 % $|\pm15/2\rangle$	±14.9611

Supplementary Note 1 All frequency dependent ac magnetic susceptibility data were fitted using a Cole-Cole function[6] in which the susceptibility is described by

$$\chi(\nu_{\mathrm{ac}}) = \chi_{\mathrm{S}} \frac{\chi_{\mathrm{T}} - \chi_{\mathrm{S}}}{1 + (2i\pi\nu_{\mathrm{ac}}\tau)^{1-\alpha}} \tag{1}$$

where ν_{ac} is frequency of the oscillating magnetic field, χ_{T} the isothermal susceptibility, χ_{S} the adiabatic susceptibility, τ the relaxation time (*i.e.* the spin-lattice relaxation time T_1 if the spin system is at thermal equilibrium with the phonon bath), and α the relaxation time distribution parameter. The latter parameter can take on values of $0 \leq \alpha < 1$, where $\alpha = 0$ corresponds to a single relaxation time. Supplementary Equation (1) can be rewritten into $\chi(\nu_{\mathrm{ac}}) = \chi'(\nu_{\mathrm{ac}}) + i\chi''(\nu_{\mathrm{ac}})$, where the real (in-phase) and imaginary (out-of-phase) components are given by

$$\chi'(\nu_{\mathrm{ac}}) = \chi_{\mathrm{S}} + (\chi_{\mathrm{T}} - \chi_{\mathrm{S}}) \frac{1 + (2\pi\nu_{\mathrm{ac}}\tau)^{1-\alpha}\sin(\pi\alpha/2)}{1 + 2(2\pi\nu_{\mathrm{ac}}\tau)^{1-\alpha}\sin(\pi\alpha/2) + (2\pi\nu_{\mathrm{ac}}\tau)^{2-2\alpha}} \tag{2}$$

$$\chi''(\nu_{\mathrm{ac}}) = (\chi_{\mathrm{T}} - \chi_{\mathrm{S}}) \frac{(2\pi\nu_{\mathrm{ac}}\tau)^{1-\alpha}\cos(\pi\alpha/2)}{1 + 2(2\pi\nu_{\mathrm{ac}}\tau)^{1-\alpha}\sin(\pi\alpha/2) + (2\pi\nu_{\mathrm{ac}}\tau)^{2-2\alpha}} \tag{3}$$

where in our case, $\tau = T_1$.

Supplementary Note 2 In the expression used to describe the temperature dependence associated with the Orbach relaxation process (second term of Equation (2) in the main text), the parameter M_O^2 appears. This parameter reflects the magnitude of the matrix elements of the dynamic crystal field potential relevant for the individual steps of the relaxation process. By combining the notation of Bierig, Weber, and Warshaw[7] and Larson and Jeffries[8], M_O^2 is defined as

$$M_{\mathrm{O}}^2 = \frac{\sum_{k,q} \left|\left\langle a \middle| \hat{V}_k^q \middle| c \right\rangle\right|^2 \sum_{k,q} \left|\left\langle c \middle| \hat{V}_k^q \middle| b \right\rangle\right|^2}{\sum_{k,q} \left|\left\langle a \middle| \hat{V}_k^q \middle| c \right\rangle\right|^2 + \sum_{k,q} \left|\left\langle c \middle| \hat{V}_k^q \middle| b \right\rangle\right|^2} \tag{4}$$

where $|a\rangle$, $|c\rangle$, and $|b\rangle$ are the initial, intermediate, and final CF states, respectively, and $\hat{V}_k^q = b_k^q \hat{O}_k^q$. $\hat{O}_k^q$ are the Stevens equivalent operators with associated dynamic crystal field parameters b_k^q. For a Kramers ion like Dy^{3+}, where $|a\rangle$ and $|b\rangle$ constitute the two components of the ground doublet, the excited state $|c\rangle$ will have a time-reversed state $|d\rangle$ constituting the other component of the excited doublet. For this reason, Supplementary Equation (4) should in principle contain an extra term with $|d\rangle$ substituting for $|c\rangle$. However, as the terms are equal, the contribution can be accounted for by a factor of two[8], which has been included in Equation (2) of the main text. In the phenomenological approach developed by Orbach, the thermal deformation of the crystal field is possibly of such low symmetry that all b_k^q might be non-zero[8]. For various salts (*e.g* the double nitrates $Ln_2Mg_3(NO_3)_{12}{\cdot}24H_2O$, and the lanthanide ethyl sulfates $Ln(EtSO_4)_3{\cdot}9H_2O$) empirical normalizing factors have been derived for estimating the values of b_k^q from the experimentally determined diagonal k'th order static parameters[8], *i.e.* $|b_k^q| = g_k^{|q|}|B_k^0|_{\mathrm{exp}}$. The values of $g_k^{|q|}$ are generally different for different values of $|q|$, but of the same order of magnitude. We shall refrain from any estimate of the b_k^q parameters in our case. However, it is worth pointing out, that the difference in the main component of the first excited state for **1Dy** and **2Dy** ($|\pm 9/2\rangle$ and $|\pm 13/2\rangle$, respectively) changes the relevant terms in the dynamic crystal field potential. For **1Dy**, the $\Delta m_J = 1$ difference between *e.g.* the $|+11/2\rangle$ component of the ground doublet and the $|+9/2\rangle$ first excited doublet renders the $b_k^{\pm 1}\hat{O}_k^{\pm 1}$ terms (k = 2, 4, and 6) relevant. The $\Delta m_J = 10$ difference between the $|+9/2\rangle$ state and the $|-11/2\rangle$ component of the ground doublet necessitates the inclusion of the $b_6^{\pm 5}\hat{O}_6^{\pm 5}$ terms. The expected dominant dynamic crystal field potential should therefore be given by

$$\sum_{k,q} \hat{V}_k^q = b_2^{\pm 1}\hat{O}_2^{\pm 1} + b_4^{\pm 1}\hat{O}_4^{\pm 1} + b_6^{\pm 1}\hat{O}_6^{\pm 1} + b_6^{\pm 5}\hat{O}_6^{\pm 5} + \cdots \ . \tag{5}$$

For **2Dy** on the other hand, the $\Delta m_J = 12$ difference between the $|+13/2\rangle$ state and the $|-11/2\rangle$ component of the ground doublet necessitates the inclusion of the $b_6^{\pm 6}\hat{O}_6^{\pm 6}$ terms rather than the $b_6^{\pm 5}\hat{O}_6^{\pm 5}$ terms relevant for **1Dy**. For this reason, the expected dominant dynamic crystal field potential for **2Dy** should therefore be given by

$$\sum_{k,q} \hat{V}_k^q = b_2^{\pm 1}\hat{O}_2^{\pm 1} + b_4^{\pm 1}\hat{O}_4^{\pm 1} + b_6^{\pm 1}\hat{O}_6^{\pm 1} + b_6^{\pm 6}\hat{O}_6^{\pm 6} + \cdots \ . \tag{6}$$

As outlined above, the g_6^5 and g_6^6 normalizing factors connecting the dynamic CF parameters to the diagonal static sixth order parameter is expected to be of the same order of magnitude but different. As the dynamic sixth order CF parameters furthermore are related to the determined values of B_6^0, the observed difference in the zero field values of M_O^2 is the expected result.

Supplementary Note 3 Calculations of the tunnel splittings were carried out within the framework of Prokof'ev-Stamp theory[9–12]. In the low-temperature regime above T_N, weak spin-spin interactions will give rise to a distribution of dynamic dipolar fields at each dysprosium site. The distribution width can be estimated from the previously determined Néel temperatures according to[11]

$$\sigma_{\mathrm{dip}} \approx \frac{k_B T_N}{2 g_J \mu_B J}. \tag{7}$$

For $g_J = 4/3$ and $J = 15/2$, values of $\sigma_{\mathrm{dip}} = 4.8$ mT and $\sigma_{\mathrm{dip}} = 3.7$ mT are calculated for **1Dy** and **2Dy**, respectively. The dipolar fields cause a splitting of the ground state of $\Delta E = (\xi_{\mathrm{dip}}^2 + \Delta_T^2)^{1/2}$. ξ_{dip} is the dipolar energy bias, and Δ_T is the tunnel splitting in the ground state. The magnitude of the separation between the ground state and the first excited state, justifies an effective $J_{\mathrm{eff}} = ½$ treatment in the temperature regime relevant for tunneling (at $T = 5$ K, the population of the first excited doublet is 0.1 ‰ and 0.2 ‰ for **1Dy** and **2Dy**, respectively). Our crystal field model affords the following components of the effective axial ground state **g**-tensors: **1Dy**, $g_{||} = 14.38$, $g_c = 0.226$; **2Dy**, $g_{||} = 14.53$, $g_{\perp} = 0.081$. By estimating the dipolar field experienced at a given site to be isotropic and estimated by the distribution width (*i.e.* $B_{\mathrm{dip},||} = B_{\mathrm{dip},\perp} \sim \sigma_{\mathrm{dip}}$)[11] the dipolar bias can be calculated according to

$$\xi_{\mathrm{dip}} = g_{||}\mu_B B_{\mathrm{dip},||} \approx g_{||}\mu_B \sigma_{\mathrm{dip}} \tag{8}$$

while the tunnel spitting is calculated as

$$\Delta_T = g_{\perp}\mu_B B_{\mathrm{dip},\perp} \approx g_{\perp}\mu_B \sigma_{\mathrm{dip}}. \tag{9}$$

The values calculated for **1Dy** are $\xi_{\mathrm{dip}} = 0.032$ cm^{-1} and $\Delta_T = 5.1 \cdot 10^{-4}$ cm^{-1}, while $\xi_{\mathrm{dip}} = 0.025$ cm^{-1} and $\Delta_T = 1.4 \cdot 10^{-4}$ cm^{-1} are obtained for **2Dy**. The calculated ground state tunnel splitting for the lower symmetry derivative **1Dy** is almost four times larger than the one in **2Dy**. If the distribution of dipolar bias is normally distributed around zero bias, *i.e.*[11]

$$P(\xi_{\mathrm{dip}} = 0) = P(B_{\mathrm{dip},||} = 0)\frac{1}{g_{||}\mu_B} = \frac{1}{\sqrt{2\pi}\sigma_{\mathrm{dip}}}\frac{1}{g_{||}\mu_B} = \frac{1}{\sqrt{2\pi}g_{||}\mu_B\sigma_{\mathrm{dip}}} \tag{10}$$

then the average incoherent tunneling rates (the dipolar fields fluctuate randomly in time, hence there is no coherence between successive tunneling events) can be calculated as[10,11]

$$\Gamma = \frac{\Delta_T^2}{\hbar} P(\xi_{\mathrm{dip}} = 0). \tag{11}$$

The calculated tunnel rates are $\Gamma = 6.0 \cdot 10^5$ s^{-1} and $\Gamma = 5.9 \cdot 10^4$ s^{-1} for **1Dy** and **2Dy**, respectively. Both rates are significantly larger than the observed ones (**1Dy**: 327(2) s^{-1}, **2Dy**: 63(4) s^{-1}). However, considering the Ising nature of dysprosium spins at low temperatures and the anisotropic crystal packings (see Supplementary Fig. 26) it is physically reasonable to allow the dipolar fields to be anisotropic ($B_{\mathrm{dip},||} > B_{\mathrm{dip},\perp}$) but to retain their overall magnitude, *i.e.* $B_{\mathrm{dip}} = (B_{\mathrm{dip},||}^2 + 2B_{\mathrm{dip},\perp}^2)^{1/2} \approx (3\sigma_{\mathrm{dip}}^2)^{1/2}$. Within such a picture it is possible to calculate the values of $B_{\mathrm{dip},\perp}$ (and therefore the tunnel splitting) which satisfy the experimentally determined tunnel rates using the relation

$$\Gamma = \frac{(g_{\perp}\mu_B B_{\mathrm{dip},\perp})^2}{\hbar}\frac{1}{\sqrt{2\pi}g_{||}\mu_B\sqrt{B_{\mathrm{dip}}^2 - 2B_{\mathrm{dip},\perp}{}^2}}. \tag{12}$$

For **1Dy**, $\Gamma \sim 3 \cdot 10^2$ s^{-1} is obtained for $B_{\mathrm{dip},\perp} = 0.15$ mT (and therefore $B_{\mathrm{dip},||} = 8.3$ mT), corresponding to $\Delta_T \sim 1.6 \cdot 10^{-5}$ cm^{-1} (and $\xi_{\mathrm{dip}} \sim 0.056$ cm^{-1}). For **2Dy**, $\Gamma \sim 60$ s^{-1} is obtained for $B_{\mathrm{dip},\perp} = 0.16$ mT (and therefore $B_{\mathrm{dip},||} = 6.4$ mT), corresponding to $\Delta_T \sim 6.1 \cdot 10^{-6}$ cm^{-1} (and $\xi_{\mathrm{dip}} \sim 0.043$ cm^{-1}). This suggests that the true increase in the size of the tunnel splitting is approximately threefold, rather than the approximately fourfold one suggested from the calculations based on isotropic dipolar fields.

Supplementary Note 4 For tunnel splittings of the determined size, the thermalization of the individual dysprosium electronic spins cannot be driven by direct spin-phonon relaxation. For a direct process, $T_1^{-1}(T) \sim \omega^5\coth(\hbar\omega/k_BT)$, and for $\omega = \Delta_T/\hbar \sim 1$ MHz, the SLR rate becomes unphysically small[11]. In addition, the phonon density at such frequencies is negligible. As the Curie-Weiss analyses of the isothermal susceptibilities suggest the spins to be at thermal equilibrium with the lattice approximately down to the ordering temperatures (see Supplementary Figure 23), an alternative, more efficient SLR mechanism must be at play. The phonon laser effect has been proposed to be such a mechanism[13]. Within such a picture, relaxation to the phonon bath is achieved through the collective generation of a transverse phonon of a frequency corresponding to the total splitting of the ground state. The maximum phonon laser rate for a given system can be calculated according to[13]

$$\Gamma_{\mathrm{L,max}} = J\sqrt{\frac{\Delta_{\mathrm{T}}^2\omega_0}{8\hbar\rho V v_{\mathrm{t}}^2}} \tag{13}$$

where J is the total angular momentum (J = 15/2 for Dy^{3+}), $\omega_0 = (\Delta_T^2 + \xi_{dip}^2)^{1/2}/\hbar$, ρ the crystal density, V the unit cell volume, and v_t is the speed of the transverse phonon. By using the values of ρ and V obtained from the single crystal structures[1] and by setting $v_t = v_m$, maximum laser rates of $\Gamma_{L,max} \sim 6\cdot10^2$ s^{-1} s^{-1} and $\Gamma_{L,max} \sim 2\cdot10^2$ s^{-1} are obtained for **1Dy** and **2Dy**, respectively. It is worth noting that frequency of the emitted phonon (ω_0) within the above outlined picture is much more on par with phonon frequencies of non-negligible density in molecular crystals (**1Dy**: ω_0 = 11 GHz, **2Dy**: ω_0 = 8.2 GHz), as compared to the $\omega \sim 1$ MHz frequencies associated with direct spin-phonon relaxation.

Supplementary References

1. Sørensen, M. A. *et al.* Imposing high-symmetry and tuneable geometry on lanthanide centres with chelating Pt and Pd metalloligands. *Chem. Sci.* **8,** 3566–3575 (2017).

2. Pedersen, K. S. *et al.* Design of Single-Molecule Magnets: Insufficiency of the Anisotropy Barrier as the Sole Criterion. *Inorg. Chem.* **54,** 7600–7606 (2015).

3. Ventura, G. & Perfetti, M. *Thermal Properties of Solids at Room and Cryogenic Temperatures* (Springer Netherlands, 2014).

4. Zadrozny, J. M. *et al.* Slow magnetization dynamics in a series of two-coordinate iron(II) complexes. *Chem. Sci.* **4,** 125–138 (2013).

5. Soeteman, J., Bevaart, L. & van Duyneveldt, A. J. The direct and Raman spin-lattice relaxation process in $YbCl_3 \cdot 6H_2O$. *Physica* **74,** 126–134 (1974).

6. Cole, K. S. & Cole, R. H. Dispersion and Absorption in Dielectrics I. Alternating Current Characteristics. *J. Chem. Phys.* **9,** 341–351 (1941).

7. Bierig, R. W., Weber, M. J. & Warshaw, S. I. Paramagnetic Resonance and Relaxation of Trivalent Rare-Earth Ions in Calcium Fluoride. II. Spin-Lattice Relaxation. *Phys. Rev.* **134,** A1504-1516 (1964).

8. Larson, G. H. & Jeffries, C. D. Spin-Lattice Relaxation in Some Rare-Earth salts. I. Temperature Dependence. *Phys. Rev.* **141,** 461–478 (1966).

9. Prokof'ev, N. V. & Stamp, P. C. E. Low-Temperature Quantum Relaxation in a System of Magnetic Nanomolecules. *Phys. Rev. Lett.* **80,** 5794–5797 (1998).

10. Wernsdorfer, W. *et al.* Observation of the Distribution of Molecular Spin States by Resonant Quantum Tunneling of the Magnetization. *Phys. Rev. Lett.* **82,** 3903–3906 (1999).

11. Luis, F. *et al.* Spin-lattice relaxation via quantum tunneling in an Er^{3+}-polyoxometalate molecular magnet. *Phys. Rev. B* **82,** 060403 (2010).

12. Martínez-Pérez, M. J. *et al.* Gd-Based Single-Ion Magnets with Tunable Magnetic Anisotropy: Molecular Design of Spin Qubits. *Phys. Rev. Lett.* **108,** 247213 (2012).

13. Chudnovsky, E. M. & Garanin, D. A. Phonon Superradiance and Phonon Laser Effect in Nanomagnets. *Phys. Rev. Lett.* **93,** 257205 (2004).
